# A transcriptome-based approach to identify functional modules within and across primary human immune cells

**DOI:** 10.1371/journal.pone.0233543

**Published:** 2020-05-29

**Authors:** Saraï Mola, Sylvain Foisy, Gabrielle Boucher, François Major, Claudine Beauchamp, Mohamad Karaky, Philippe Goyette, Sylvie Lesage, John D. Rioux

**Affiliations:** 1 Centre de recherche, Institut de cardiologie de Montréal, Montréal, Québec, Canada; 2 Unité de recherche en ingénierie des ARN, Institut de recherche en immunologie et en cancérologie, Montréal, Québec, Canada; 3 Département d’informatique et de recherche opérationnelle, Université de Montréal, Montréal, Québec, Canada; 4 Département de biochimie et médecine moléculaire, Université de Montréal, Montréal, Québec, Canada; 5 Centre de recherche, Hôpital Maisonneuve-Rosemont, Montréal, Québec, Canada; 6 Département de microbiologie, infectiologie et immunologie, Université de Montréal, Montréal, Québec, Canada; Centro Cardiologico Monzino, ITALY

## Abstract

Genome-wide transcriptomic analyses have provided valuable insight into fundamental biology and disease pathophysiology. Many studies have taken advantage of the correlation in the expression patterns of the transcriptome to infer a potential biologic function of uncharacterized genes, and multiple groups have examined the relationship between co-expression, co-regulation, and gene function on a broader scale. Given the unique characteristics of immune cells circulating in the blood, we were interested in determining whether it was possible to identify functional co-expression modules in human immune cells. Specifically, we sequenced the transcriptome of nine immune cell types from peripheral blood cells of healthy donors and, using a combination of global and targeted analyses of genes within co-expression modules, we were able to determine functions for these modules that were cell lineage-specific or shared among multiple cell lineages. In addition, our analyses identified transcription factors likely important for immune cell lineage commitment and/or maintenance.

## Introduction

The human immune system protects us from microbes (bacteria, viruses, fungi, and parasites) that penetrate the physical and chemical barriers of the body. It fulfills surveillance functions in order to detect and eliminate aberrant cells that result from infection, cancer, and senescence. In contrast, the immune system is also at the heart of pathogenic syndromes and chronic diseases that involve virtually all organ systems (e.g. cardiovascular, gastrointestinal, respiratory, musculoskeletal), and are associated with significant morbidity and mortality [1–5].

Given the central importance of the immune system in human health and disease, extensive work by numerous research groups over the past 100+ years has provided a detailed functional understanding of the cells and organs within the immune system. This includes how the multiple different cell populations, which make up the complex immune system, differentiate from a common pluripotent progenitor cell, how different cells communicate through an extensive system of cytokines, chemokines, and associated receptors, and how signaling pathways can regulate cell response to various stimuli [6–8]. This functional understanding has been made possible by a combination of human and animal model systems, sensitive and specific reagents, such as antibodies directed against cell surface and intracellular proteins, and the development of technological platforms to isolate and study-specific cell populations based on multi-parameter flow cytometry, fluorescence-activated cell sorting, and mass cytometry [5, 9].

Genome-wide transcriptomic-based approaches have been instrumental in the identification of molecular classifiers of leukemias, interferon and granulopoiesis signatures in the blood of autoimmune patients, cytokine response pathways of T cells in psoriasis and the activation of macrophages, to name a few [10–12]. Transcriptomic analyses have also been employed to perform surveys of the different cell populations within the myeloid and lymphoid lineages of the mouse immune system in different contexts (e.g. age, differentiation and activation states, tissue location) [13, 14]. More recently, the use of single-cell RNA sequencing to evaluate the transcriptome has enabled the identification of previously unrecognized rare cell subsets of the human immune system [15–18]. Together, these studies suggest that genome-wide transcriptomics is an important tool for studying how the human immune system responds to different conditions (e.g. stimuli, genotypes, health status, response to therapy).

The identification of gene sets that robustly represent different conditions has the potential to become powerful biomarkers, even without fully understanding the impact of the observed transcriptomic changes. The functional interpretation of changes in transcriptome between different conditions, however, remains an important challenge. A common approach to addressing this problem is to first perform gene co-expression analyses, for identifying gene sets that potentially are involved in common biological functions, followed by annotation-based analyses (e.g. enrichment of annotation terms, guilt-by-association) for attributing specific functions to the observed gene sets [19–21]. The success of this approach depends on numerous factors such as the complexity of the samples being tested, the true extent of the differences in the transcriptomes between the different conditions, the degree of experimental variability in the collection, processing, and sequencing of the samples [19–21].

In the current study, we were interested in testing this approach in the context of circulating human immune cells because of the well-characterized and distinctive nature of the different immune cells in terms of cell-specific markers (and thus genes expressed) and functions, as well as the relative ease by which highly purified immune cell populations can be isolated from peripheral blood. Specifically, we analyzed the transcriptomes of nine distinct immune cell populations from the peripheral blood of twelve healthy volunteers using a combination of global and targeted approaches to functionally annotate modules of co-expressed genes. We were able to attribute functions to most of the modules that were either cell population-specific or shared across more than one cell population. In addition, we identified TFs associated with lymphoid vs. myeloid lineages, T and B cell fates, many of which were previously known to be involved in lineage differentiation, as well as others that we propose as new candidates for having a role in lineage commitment and/or lineage maintenance.

## Materials and methods

### Subject selection criteria, ethics committee approval, and blood sample procedures

The project was submitted to and approved by the MHI Institutional Ethics Committee «Comité d’éthique de la recherche et du développement des nouvelles technologies ». Volunteers were then solicited via postings across the Montreal Heart Institute (MHI) and directed to the MHI Biobank Center for screening, informed consent form completion, and venipuncture. To be selected, the following criteria were used: (1) adult males with no history of (or ongoing) chronic inflammatory disease; (2) no current use of anticoagulants; (3) no use of oral steroids or cyclosporine in previous four weeks; (4) no use of NSAIDs in previous three days. After signing the informed consent form, volunteers were assigned a randomly generated ID number by the MHI Biobank Center; they were then called for blood withdrawal according to the availability of the cell sorter platform. The assigned ID number was provided with the blood samples to ensure anonymity. We recruited 12 individuals over a period of nine months. Researchers can request access to the individual-level data from the current study by contacting the Montreal Heart Institute ethics committee at the following institutional email address: cer.icm@icm-mhi.org.

### Blood collection and cellular isolation

For lymphocytes and monocytes isolation, blood was collected in Vacutainer CPT Mononuclear Cell Preparation Tube—Sodium Citrate (BD Biosciences, San Jose, CA). Centrifugation was performed according to the manufacturer's instructions. Mononuclear cells were collected and washed three times in saline buffer, then filtered through a 70um mesh cell strainer. The cells were further purified with anti-CD14 coated microbeads using LS columns and the QuadroMACS separator stand (Miltenyi, Cambridge, MA), according to the manufacturer's instructions. Non-specific binding of antibodies was prevented on both CD14+ and CD14- enriched fractions by using FcR blocking reagent (Miltenyi). A portion of each fraction was used for immunophenotyping.

The CD14+ enriched mononuclear fraction was stained with a goat anti-mouse IgG2a antibody (Serotec, Kidlington, United Kingdom). CD14- enriched mononuclear fraction was stained with antibodies against CD3 (clone UCHT1), CD4 (clone RPA-T4), CD8 (clone RPA-T8), CD19 (clone HIB19), TCRγδ (clone B1) (Biolegend, San Diego, CA) and CD56 (clone B159, BD Biosciences). Monocytes, CD4+ T cells (CD3+CD4+CD19-TCRγδ-), CD8+ T cells (CD3+ CD8+CD19-TCRγδ-), TCRγδ T cells (CD3+CD19-TCRγδ+), NK cells (CD3-CD19-CD56+) and B cells (CD3-CD19+) were sorted on a FACSAria™ III cell sorter (BD Biosciences) based on cell size, granularity, doublet exclusion, live/dead marker and surface expression of targeted molecules. Purity was > 94% (median 98.1% with 97.3% and 98.9% for the 1st and 3rd quartiles, respectively).

For neutrophil isolation, blood was collected in Vacutainer® EDTA collection tubes (BD Biosciences). CD15+ fraction was enriched with StraightFrom™ Whole Blood CD15 MicroBeads using Whole Blood columns with the QuadroMACS separator (Miltenyi), according to the manufacturer's instructions. Non-specific binding of antibodies was prevented on the enriched CD15+ fraction by using FcR blocking reagent (Miltenyi). Cells were stained for CD14 (clone M5E2) and CD16 (clone 3G8) (Biolegend). Neutrophil population (CD14-CD16+) was sorted on a FACSAria™ III cell sorter (BD Biosciences) based on cell size, granularity, doublet exclusion, live/dead marker and surface expression of targeted molecules. Purity was > 99%.

### Macrophage differentiation and activation

CD14+ enriched mononuclear cells were frozen in the vapor phase of liquid nitrogen until the day of culture. After thawing, the cells were cultured at 37°C 5% CO_2_ in RPMI 1640 medium with GlutaMAX™, 10% heat-inactivated fetal bovine serum, 100 units/mL penicillin, 100 μg/mL streptomycin (ThermoFisher Scientific, Mississauga, Canada) and 50ng/ml M-CSF (Millipore, Etobicoke, Canada) for a total of 8 days, with media renewal every 2 days, to obtain macrophages. Activated macrophages were also generated by stimulating the macrophage cultures with 1ug/ml LPS (Sigma, Oakville, Canada) during the last 24h of culture.

### Immunophenotyping

Following FcR blocking reagent (Miltenyi) step, CD14+ and CD14- enriched mononuclear cells were stained with antibodies described in **S1 Table**. *In vitro* generated macrophages were blocked with FcR blocking reagent (Miltenyi) and stained with antibodies listed in **S2 Table**. Cells were analyzed on a BD™ LSR II flow cytometer (BD Biosciences). Final immunophenotyping results were generated using FlowJo software (FlowJo LLC, Ashland, OR) according to size, granularity, live/dead marker and surface expression of targeted molecules. Results are summarized in **S1 Fig**.

### RNA extraction and sequencing

The total number of samples obtained for each population from each individual is summarized in **S3 Table**. Total RNA from all cell samples was isolated using a Qiagen RNeasy Plus Mini kit (Qiagen, Toronto, Canada) with an additional step of RNase-free DNase set (Qiagen) DNaseI digestion; quantification, as well as quality control, were performed on Agilent BioAnalyzer 2100 (Agilent, Santa Clara, CA) using an Agilent RNA 6000 Nano Kit, and aliquots for sequencing were prepared and kept at -80°C until further processing.

RNA sequencing was performed at McGill/Genome-Québec Innovation Center. Total RNA was extracted and all, but one sample had RNA integrity number (RIN) greater than 8 (RIN ranged between 8.4 and 10; the one sample with RIN<8 was excluded). RNA samples were transformed into barcoded DNA libraries using Truseq Stranded mRNA library preparation kits (Illumina, San Diego, CA). Paired-end sequencing, generating 2x100bp reads, was performed on an Illumina HiSeq2000 with raw FASTQ sequences downloaded from the platform’s server for local pre-processing and analysis.

### Bioinformatic processing of sequence files

Raw FASTQ formatted sequence files were collected from the McGill/Genome-Québec Innovation Center sequence service. Primary QC analysis was performed using FastQC (v0.10.1) (https://www.bioinformatics.babraham.ac.uk/projects/fastqc). Following a manual inspection of all FastQC result files to check for low-quality samples, raw sequence files were trimmed of low-quality bases from reads and removing low-quality reads altogether as well as removing potential adapter contamination with Trimmomatic (v0.33) [22] using a modified adapter FASTA file with the following parameters: ILLUMINACLIP with seed mismatches set to 2, palindrome clip threshold set to 30 and simple clip threshold set to 15, TRAILING set to 20 and MINLEN set to 50. Quality- and adapter-trimmed FASTQ files were inspected again with FastQC to confirm global quality.

Alignments were performed with TopHat 2 (v2.1.0) [23] using iGenome index and annotation files built from build 38 of the human genome (Illumina). Subsequent filtering of the aligned reads was done using SAMTools (v1.6) [24] to keep only properly-paired and single-mapped reads in the final alignments. Summary statistics from all steps for each cell type are shown in **S3 Table**.

For gene expression analysis, R/Bioconductor was used with a variety of additional analysis modules. Alignment files were processed with easyRNASeq (v1.8.8) [25] to measure gene abundance and create the gene expression matrix of raw read counts that was further processed using edgeR (v3.12.1) [26, 27] and Limma (v3.26.9) [28]. edgeR package was used to generate the expression count matrix and annotations with DGEList function and to filter out non- and low-expressed genes based on a cpm threshold. edgeR was also used to obtain per sample normalization factors. The function voom from the Limma package was used to normalize data from gene expression for library sizes scale with TMM factors and then to stabilize variance in the data. The various cell types collected in the study have expression profiles (e.g. neutrophils) with too much dissimilarity to allow for a normalization approach based on the hypothesis of relatively similar expression, for example, the normalization by quantile (**S2 Fig**) [29].

### Principal component and hierarchical clustering analyses

**Fig 1:**
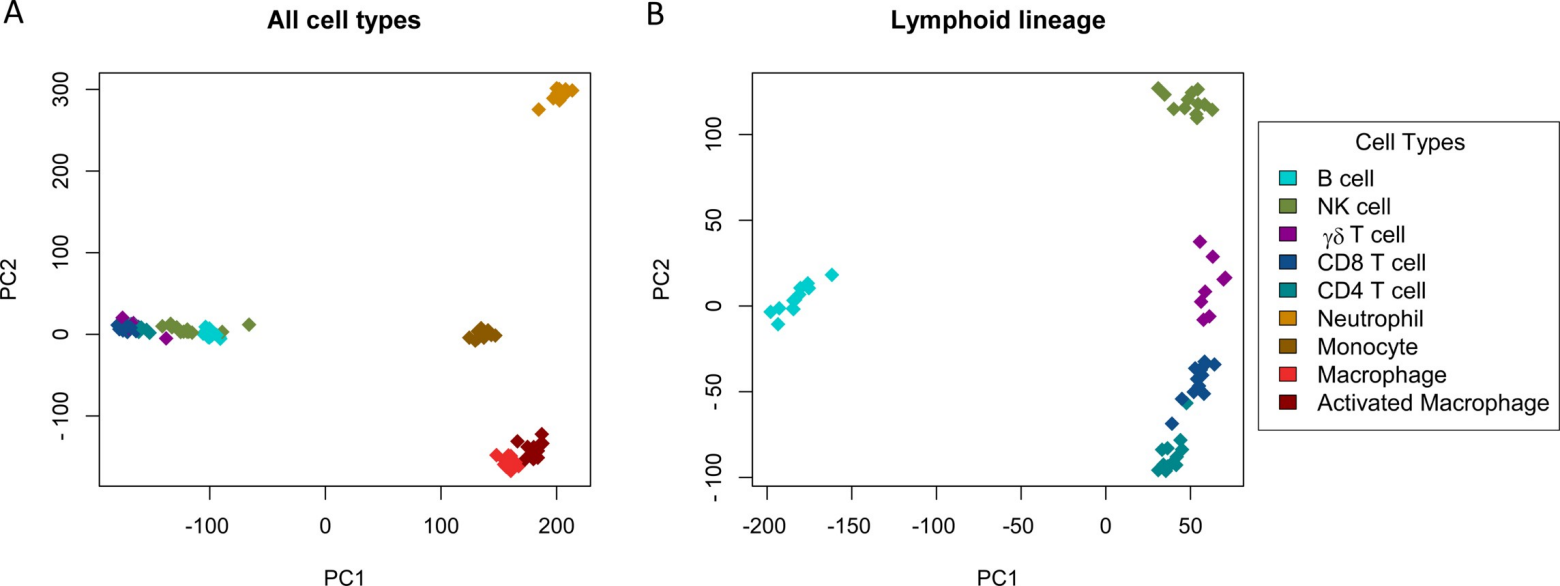
Transcriptomic data distinguish immune cell types. Representation of the first two principal components on RNA-seq data from all individuals for all samples (Panel A) and for lymphoid samples alone (Panel B). The R function *prcomp* was used to perform principal component analysis, and each symbol represents an individual RNA-seq sample.

**Fig 2:**
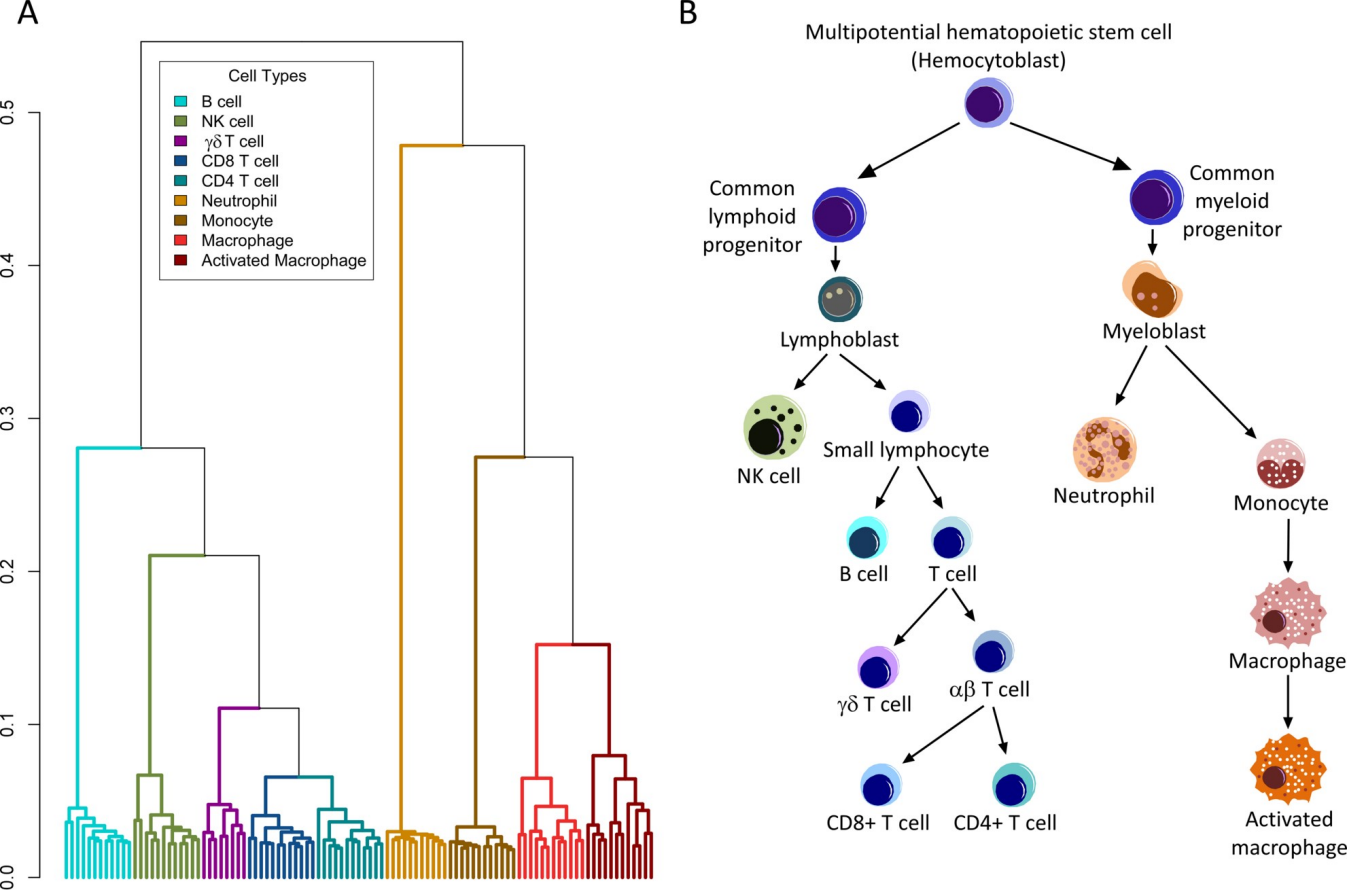
Unsupervised hierarchical clustering of RNA-seq data reflects hematopoietic differentiation. A dendrogram representation of the unsupervised hierarchical clustering of RNA-seq data from across all individuals and cell types (Panel A) has a structure that closely resembles the known differentiation scheme of immune cells (Panel B). Each dendrogram leaf represents a single RNA-seq sample and Y-axis represents the distance based on Pearson correlation. Black arrows represent a decision in the known differentiation scheme of immune cells.

Analyses of transcriptomic data using Principal Component Analysis (PCA) and hierarchical clustering approaches were performed to provide a global view of the transcriptomic data. These approaches enabled us to identify similarities between samples and structure in the dataset. This allowed a first assessment of the underlying principal sources of variation. After filtration and normalization, principal components were computed on all detectable transcripts with *prcomp* in R thus representing multiplicative differences between samples. The first few PCs [30–32] were investigated because they represented large axes of variation, using graphical display (**Fig 1**). In complementary, unsupervised hierarchical clustering was used to investigate the grouping of all samples. For this analysis on the entire normalized transcriptome, Pearson correlation was chosen to evaluate the distance between samples with R base functions, *cor*, and *dist*. Hierarchical clustering was used to build our data dendrogram with *hclust* R function (**Fig 2A**). Finally, ANOVA was used to determine the percent of gene expression variance explained by cell types. The expression for each gene was centered and scaled to a mean and variance of respectively 0 and 1. Analysis of variance was performed on each gene to obtain the sum of squares (SS) explained by cell types. Multivariate variance explained was estimated by adding explained SS from all genes and then dividing by the total SS.

### Definition of co-expression modules

Starting with the hypothesis that genes with a similar function will often be co-expressed, we built co-expression modules with the R package WGCNA (v1.64–1) using the RNA-Seq data from our nine primary immune cell types. The objective of this tool is to cluster genes based on similar expression profiles between samples [33, 34]. For building co-expression networks with WGCNA, we choose a signed topology to compute gene co-expression similarity. Signed topology makes it easier for interpretation purposes or relevant biology, such as making distinction gene repression and activation [19]. A connectivity matrix between genes was then computed that describes how strongly genes are connected to all others. For that purpose, a “soft thresholding” parameter β must be chosen [34]. This parameter is an exponent to the correlation matrix that determines the emphasis put on higher vs lower correlations. The *pickSoftThreshold* function was used to help in choosing β. After analysis of network topology for various β values, a value of 12 was selected as a good trade-off between scale-free topology and connectivity (**S3 Fig**). Therefore, to compute dissimilarities between genes, we used the WGCNA functions *adjacency* and *TomsimilarityFromExpr* (Topology Overlap Matrix Similarity From Expression) with the following parameters: Pearson correlation, signed topology and a β of 12. Based on the TOM dissimilarity measure, *hclust* function was used to build a hierarchical clustering of the genes. Finally, *cutreeDynamic* function with minClusterSize and deepSplit parameters to 20 and 2, respectively [35], was used to cut the hierarchical tree into gene modules (**S4 Fig**). To merge closely related modules among our 63 modules, *MergeCloseModules* function was applied with cutHeight parameter of 0.05. Each module was represented by its first principal component using the function *moduleEigengenes*. Correlation and corresponding p-values of association between a given module and a cell type were then evaluated from these principal components using *cor* and *corPvalueStudent* functions from the WGCNA package.

### Global and targeted functional annotation

We used a combination of global and targeted analyses of gene annotations to identify potential biological functions represented in each module. For a global analysis of genes within each module, we used DAVID (Database for Annotation, Visualization, and Integrated Discovery) [36] to test for enrichment of functional annotations, specifically using the annotation from Gene Ontology (GO) terms for Biological Processes, Molecular Functions, and Cellular Components, Up-Keywords, Interpro and KEGG, [37–40]. In **S4 Table**, we report all annotations for which the enrichment was ≥ two-fold and a *P* <0.05.

For the targeted analyses, we undertook a more detailed approach taking advantage of the information within GeneCards [41], PubMed [42] and Google Scholar [43] databases, using gene symbols as query parameters. This approach was applied to two gene lists per module; the first list consisted of the set of genes most highly expressed in each module, with the hypothesis that the highly expressed genes may provide insight to important functions within each module. Specifically, we examined the known functions of the top 2% expressing genes (corresponding to the 98^th^ percentile) which we called these the *Top Expressing Genes* (TEGs). The second list consisted of the genes that had the most important impact on the first principal component, regardless of their level of expression. Specifically, we ranked genes based on their contribution to the first principal component of the module in question with the objective of examining the known functions for the genes that accounted for 20% of PC1, or the top 20 genes if this exceeded 20 genes, *we called these the Module Representative Genes (MRGs)*. For modules 22, 26, 38, 41, we also performed this detailed functional annotation to the entire set of genes within the modules in order to compare with the results obtained from these targeted analyses.

### Analyses of transcription factors and transcription factor binding sites in B cells

Genes within modules were annotated as TFs based on the catalog of human TFs published by Lambert and colleagues [44]. In this resource, based on a collection of databases including TRANSFAC, HT-SELEX, UniProbe, and CisBP to identify human TFs and their binding site motifs [45–54], a TF was defined as a protein capable of a) binding DNA in a sequence-specific manner and b) regulating transcription. In total, they identified 1,639 human TFs, and binding motifs for two-thirds of these (see http://humantfs.ccbr.utoronto.ca/index.php) Transcriptional control of genes within each module was analyzed in the following fashion: (1) the transcription start site (TSS) was located for each gene using the GRCh38 Ensembl database using the biomaRt tool [55]; (2) promoter regions were then defined as -1000 to +500 base pairs around these TSS; while somewhat arbitrary, this is a commonly used definition of the proximal promoter [56, 57]; and (3) these promoter regions were examined for the presence of TFBS as defined by ChIP-Seq data from the ENCODE (Encyclopedia of DNA Elements) Project [58]. As this latter data was referenced to the human genome build GRCh37, TFBSs were converted to GRCh38 version using liftOver [59]. This ENCODE dataset consisted of ChIP-Seq data for a total of 161 TFs in 80 immortalized cell lines. Of the nine immune cell populations within the current study, only the B lymphocyte population was represented in this dataset. For the modules associated with B lymphocytes, we restricted our analyses to the 76 TFs that were studied in the GM12878 immortalized B cell line. Enrichment of TFBS in the promoter regions within each of these modules was calculated with the hypergeometric distribution. Enrichment p-value threshold was 0.05.

### Transcription factors potentially associated with cell lineages and states

We examined all 1600 human TFs [44] for expression patterns across the cell types that were consistent with having a role in cell fate decisions, independent of co-expression modules described above. Following the differentiation scheme depicted in **S5 Fig**, we considered each branch point like a cell fate decision and looked for TFs that appeared to be specific for a given fate (e.g. myeloid vs. lymphoid; neutrophil vs. monocyte; B cell vs. T cell; etc.). Several TFs were very well known to be implicated in cell fate decisions (e.g. PAX5 for B cell differentiation; EOMES for differentiation of cytotoxic cells; FOXP3 for the differentiation of CD4+ T cells). Based on the expression pattern of these well-known TFs involved in differentiation of immune cells, we defined the following algorithm for prioritizing TFs potentially having a role in cell fate decisions (**S5 Fig and S5 Table**): (1) cell types resulting from a cell fate decision (e.g. lymphoid) should have an average number of read counts for the given TF at least 15-fold greater than the average number of read counts for cells from the same branch point that do not have the same fate (e.g. myeloid); (2) each cell type resulting from a cell fate decision should have an average number of read counts >100 for the given TF in order to reduce artifacts resulting from the greater variance at low read counts; (3) the cell type resulting from a cell fate decision with the lowest read count for the given TF should be at least four-fold greater than the cell type with the highest read count from the same branch point that does not have the same fate (e.g. IKZF3 in CD4+ T cells vs LPS-activated macrophage). Finally, we identify which of these candidate TFs are known to be implicated in associated cell fate decisions based on a search of the PubMed, GeneCards, Entrez, UniProt, SwissProt.

## Results

### Data generation: Isolation of immune cells and RNA-Seq analysis

For the current study, we isolated neutrophils, B cells, CD4+ and CD8+ T cells, NK cells, γδ T cells and monocytes from the peripheral blood of 12 healthy volunteers. In addition, we differentiated *in vitro* the monocytes into mature macrophages, as well as stimulated these macrophages with LPS, thus generating a total of nine different cell populations. We obtained highly pure immune cell populations, and immunophenotyping analyses confirmed the representation of subpopulations that can be expected in the circulation of healthy adults (**S1 Fig**). A transcriptomic analysis of the total RNA extracted from these cells was performed using a paired-end sequencing approach, that resulted in a very good depth of coverage (2.48x10^7^ read counts per sample on average; **S3 Table**), and thus can be expected to provide quantitative measures of transcript abundances even for less common transcripts [60]

### Structure of transcriptomic data is a reflection of the known differentiation scheme for immune cell subsets

Prior to embarking upon in-depth analyses of this RNA-Seq data, we wanted to assess whether the patterns of gene expression could potentially be correlated to differences in functions between cell populations. Principal component analysis (PCA) revealed a clear separation of the cell types on the first two components, representing the nine populations into four very distinct clusters (neutrophils, lymphocytes, monocytes, and macrophages), with all individuals clustering tightly within each cell population (**Fig 1A**). This is concordant with the observation that 86% of the total multivariate variance is between the cell types and the first two components capture a large proportion of the total variance (43% and 25% respectively). We also applied a similar PCA only on lymphoid cell types, which resulted in five distinct clusters representing the five different lymphoid populations: B cells, CD4+ and CD8+ T cells, NK cells and γδ T cells (**Fig 1B**). Hence gene expression in our data set clearly represents more differences between immune cell populations than between individuals.

Using an unsupervised hierarchical clustering we found that the dendrogram reflected the differentiation scheme of pluripotent stem cells into myeloid and lymphoid cell types, except for B and NK cells, which were interchanged in our hierarchical clustering (**Fig 2**). Given these observations, we propose that there is a great potential to identify differences in gene expression between immune cell populations that will reflect the functional biology of these populations.

### Defining potential functional groups using a combination of gene co-expression and functional annotation

**Fig 3:**
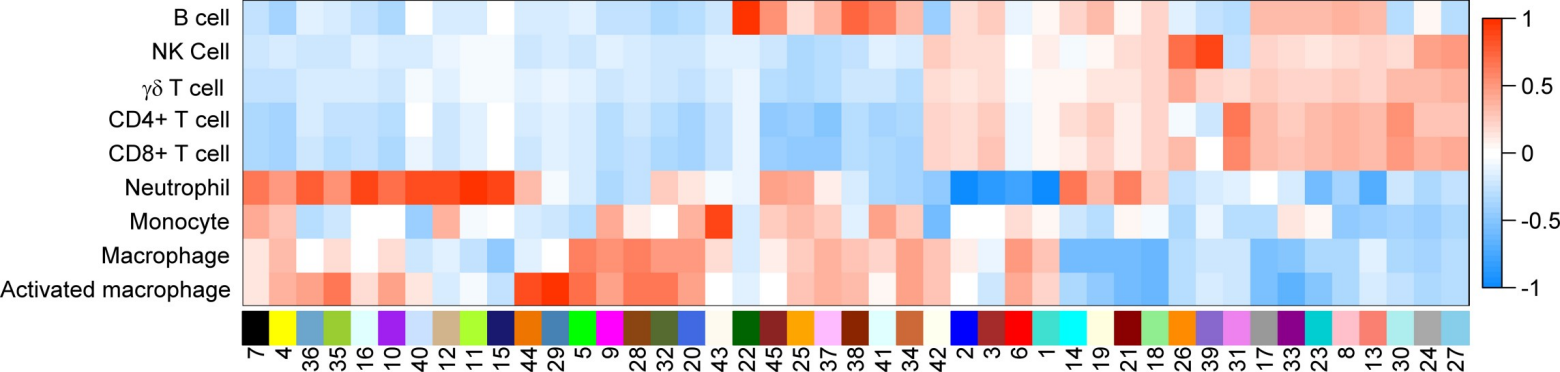
Heatmap of the correlation values of WGCNA modules with primary immune cell types. Columns represent modules computed with WGCNA and rows, primary immune cell types. Correlation values and *P* values are displayed in S6 Fig.

Knowing that there is a significant correlation structure in the transcriptome captured by the RNA expression data sets, and those co-expressed genes are often related functionally [61], we hypothesized that defining modules of co-expressed genes would be a relevant starting point for exploring potential functional units relevant to these immune cells. We, therefore, used a weighted gene co-expression network analysis (WGCNA) approach to define modules of co-expressed genes [33]. Using this approach, we obtained 45 modules with an average of 318 genes per module (ranging from 26 to 1,945 genes; **Table 1**). As can be seen in **Fig 3**, each module could be associated with one or more immune cell populations. For example, ***modules 22 and 38*** were highly associated with B cells (r = 1.00, *P* = 1.97 x10^-106^; r = 0.74, *P* = 8.05x10^-19^; respectively) and ***module 41*** with B cells and monocytes (r = 0.63, *P* = 1.41x10^-12^; r = 0.46, *P* = 8.32 x10^-07^; respectively), whereas ***module 26*** was strongly associated with NK, γδ T and CD8+ T cells (r = 0.68, *P* = 1.71 x10^-15^; r = 0.41, *P* = 1.78 x10^-05^; r = 0.32, *P* = 9.80x10^-4^; respectively), all cell populations that perform cytotoxic functions.

**Table 1:** Summary of key functions identified for modules of co-expressed genes.

Module ID	# Genes in module	Associated cell types ***									Main functions identified
		B	NK	γδ T	CD4+	CD8+	Neut	Mon	M⏀	LPS	
1	1212								0.3	0.23	Mitochondrial functions, ribosome biogenesis
2	1945										Broad range of basic functions
3	619	0.26		0.2	0.27	0.29					Transcriptional regulation via zinc finger proteins
4	593						0.48	0.32	0.35	0.39	Lysosomal function; neutrophil polarity; chemoattraction
5	489								0.62	0.69	Chemokine production, microtubule network; extracellular exosomes
6	748								0.49	0.43	Mitochondrial metabolism
7	452						0.68	0.44			Azurophil granules; cytoskeleton dynamics; ruffle
8	411	0.37	0.18	0.25	0.38	0.37					Transcriptional regulation (e.g. of cytokine genes) via zinc finger proteins
9	427							0.44	0.55	0.48	Endocytosis & ROS production
10	309						0.71			0.44	Actin remodeling & membrane trafficking, nicotinamide metabolism
11	296						0.96				Production of Azurophil granules
12	548						0.85	0.39			Cytoskeleton remodeling; cell polarity; chemokine signaling
13	401	0.33	0.22	0.23	0.32	0.32					Cell-cell adhesion; actin remodeling; interferon response
14	246	0.24					0.64				Ubiquitin-like conjugation; control of intracellular membrane traffic; transcriptional regulation
15	595						0.9				Defensin-specific granules
16	426						0.91				NET-associated histone production
17	414	0.34	0.21	0.24	0.32	0.32					Transcriptional regulations; control of immune cell proliferation
18	226	0.2	0.23	0.22	0.21	0.23	0.27				N.D.
(19)	321	0.36					0.35				Cytoskeleton remodeling
20	429							0.39	0.5	0.47	Lysosomal/Endosomal functions
21	388						0.61				Neutrophil polarization, trafficking, and exocytosis; ROS & NET production
22	195	1									Maintenance of B cell lineage; BCR signaling
23	637	0.35		0.22	0.34	0.34					Ribosomal functions
24	177		0.45	0.36	0.31	0.37					Control of Wnt signaling via beta-catenin TCF complex
25	147						0.42	0.33	0.28	0.32	Superoxide-generating NADPH oxidase activator activity
26	129		0.68	0.41		0.32					Cytotoxic granule composition; non-self-recognition
27	120		0.49	0.37	0.31	0.4					Modulation of receptor signal transduction
28	279								0.6	0.65	Bioenergetics (e.g. fatty acid oxidation, protein turnover)
29	93									0.98	Anti-microbial functions (chemokine production & zinc metabolism)
30	91			0.33	0.52	0.51					Regulation of T cell signaling
(31)	81				0.66	0.57					Modulation of immune response including via CD28, netrin 4 and semaphorin 5A
(32)	229						0.24		0.5	0.65	Vesicular trafficking (lysosomes, endosomes, exosomes)
33	67	0.34	0.18	0.23	0.26	0.28					N.D. (associated with lymphocytes)
(34)	61	0.32						0.27	0.47	0.45	Antigen presentation; vesicle function and trafficking; RAS signaling
(35)	58						0.54			0.68	Response to hypoxia; autophagy; regulation of inflammation
36	52						0.76			0.47	Endocytosis and membrane trafficking
37	46	0.39						0.28	0.36	0.36	Intracellular vesicle formation and function, including lysosomes
38	45	0.74							0.3	0.35	Regulation of BCR signaling
(39)	43		0.91	0.22							Golgi apparatus
40	121						0.88				Transcriptional regulation via zinc finger proteins
41	39	0.63						0.46	0.22		MHC class II antigen processing and presentation
42	38		0.26	0.19	0.22	0.22			0.28	0.32	Heterogeneous functions; IL7R signaling
43	35							0.92			N.D.
(44)	34						0.36			0.87	Interferon response
(45)	26	0.56					0.44	0.27			Regulation of intracellular membrane trafficking

Module ID: Module number; Module ID in parentheses: the associated functions are more speculative as enrichment is more limited; ***: only correlations between modules and cell types where *P*<0.05 are indicated; B: B cells; NK: NK cells; γδ T: γδ T cells, CD4+: CD4+ T cells; CD8+: CD8+ T cells; Neut: neutrophils; Mon: monocytes; M⏀: macrophages; LPS: LPS-activated macrophages. N.D.: Non-determine.

To gain a better understanding of the biology underlying these co-expression modules that were highly associated with one or more specific cell types, we used a combination of global and targeted analyses of gene annotations and identified potential biological functions represented in each module. For the global analysis, we tested for the enrichment of annotation terms within the Gene Ontology (GO), UniProt, InterPro and KEGG databases [37, 40]. For the targeted analysis, we undertook two independent approaches. The first was to identify the set of genes most highly expressed in each module, with the hypothesis that the highly expressed genes may provide insight into important functions within each module. Specifically, we examined the known functions of the genes that are within the top 2% of genes expressed within the cell types associated with a given module. We call these the *Top Expressing Genes* (TEGs). The second targeted approach entailed the identification of the genes with the greatest impact on the first principal component for each module, with the hypothesis that these genes better represent the modules, regardless of the expression level. Specifically, we examined the known functions of the genes that can account for 20% of the variance captured by the first principal component; We call these the *Module Representative Genes* (MRGs). To assess whether these approaches could identify functions that are relevant to the broader set of genes within each module, we also examined the known function for the entire set of genes within ***modules 22*, *38 and 41***.

### B cell-specific modules; cell activation and BCR engagements

As mentioned, ***modules 22 and 38***, are highly associated with B cells (**Fig 3**) and contain 195 and 45 genes, respectively (**Table 1**). In our global analysis of ***module 22***, there were eight annotation terms that were below 5% False Discovery Rate (FDR), and included “B-cell activation”, “Primary Immunodeficiency”, and “Immunoglobulin domain”, with a total of 17 shared genes being associated with one or more of the annotation terms mentioned previously (**S4 Table**). For ***module 38***, 16 annotation terms with *P* ≤ 0.05 were detected, but one was below 5% FDR, and none appeared to indicate functions specific to B cells (**S4 Table**).

**Fig 4:**
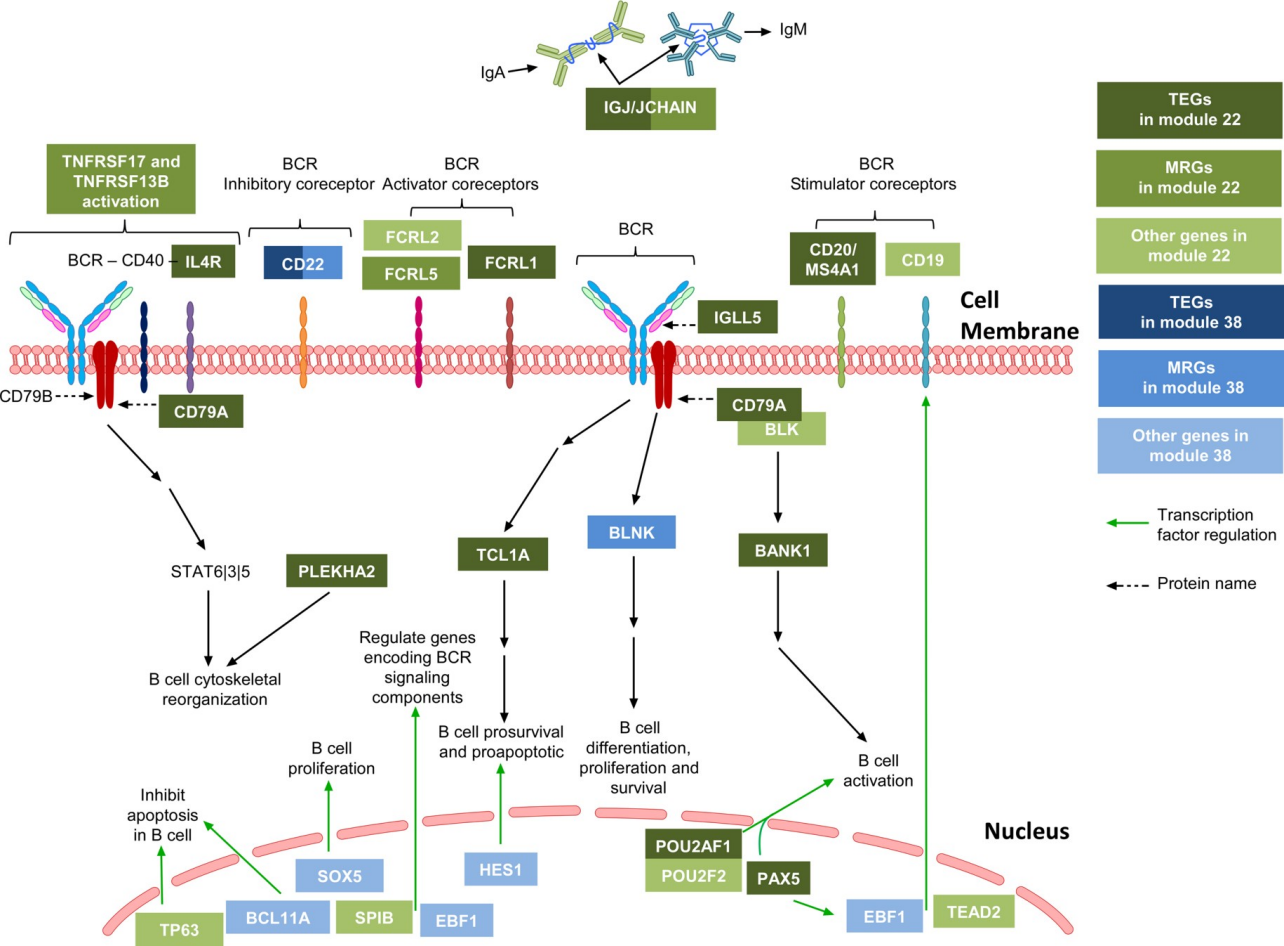
Biological functions ascertained from the analysis of genes within two B cell-associated modules. The genes within ***modules 22 and 38*** encode multiple genes that are central to B cell functions. These include components of B cell receptor (BCR) complex, such as inhibitory and stimulatory co-receptors CD22 and CD19, as well as TFs regulating the transcription of many of these. Genes in ***modules 22 and 38*** are represented in green and blue, respectively: TEGs in dark colors, MRGs in intermediate colors and other genes in light colors. To see the profile of gene expression mean of all genes of module 22 presented in this Figure refers to the heatmap in S7 Fig.

Regarding TEGs in B cells, there were 12 TEGs within ***module 22*** and three TEGs within ***module 38*** (**S6 Table**). Querying of public databases (GeneCards, PubMed and Google Scholar) for the known functions of the TEGs within ***module 22*** revealed that many are involved in B cell receptor (BCR) structure and/or signaling (e.g. *IGLL5*, *CD79A*, *BANK1*, *MS4A1*, *FCRL1*) [62]. There were also two genes encoding transcription factors (TF), *POU2AF1* and *PAX5*, associated with B cell biology [63, 64]. Interestingly, *PAX5* also regulates the expression of *EBF1*, a gene within ***module 38***, a TF crucial for B cell differentiation [65]. In the case of ***module 38***, two of the three TEGs have known functions in B cells: *CD22* inhibits BCR activation and *FCRLA* is involved in immunoglobulin assembly [66, 67] (**Fig 4**).

Regarding the MRGs, there were 28 within ***module 22*** and seven within ***module 38*** (**S6 Table**), with some overlap between the MRG and TEG lists: the *IGJ* gene (alias *JCHAIN*) encoding the immunoglobulin J chain in ***module 22*,** as well as the *CD22* and *FCRLA* genes in ***module 38*** (**S6 Table**). Among the MRGs from ***module 22*** are the *TNFRSF13B* (alias *B cell-activating factor*, *BAFF*) and *TNFRSF17* (alias *B Cell Maturation Antigen*, *BCMA*) genes, which play an important role in B cell activation. The remainder of these MRGs is poorly-characterized genes or non-protein coding RNAs (9 of 28). Of the poorly characterized genes, some are expressed in B cells (e.g. *HTR3A* [68]) or involved in B cell development/maturation (e.g. *KLHL14* and *VPREB3* [69, 70]). Others, such as *CHL1* and *TCL1A* have no known links to B cell function and merit further functional studies. Specifically, *CHL1* may be involved in cell adhesion and migration, as these functions are ascribed to its homolog *L1CAM* [71] and *TCL1A* is involved in AKT activation, which is important for BCR signaling [72].

There were five MRGs from ***module 38*** that were also not TEGs: *BLNK*, *DENND5B*, *DIRAS1*, *RASGRF1*, and *LOC102724714*. *BLNK* encodes a critical adaptor protein that bridges BCR-associated kinase activity with downstream signaling events [73, 74]. *DENND5B* is a poorly characterized gene containing a MAP kinase activating death (MADD/DENN) domain. While the function of DIRAS1 and RASGRF1 are not well understood, it is likely that these RAS family proteins are involved in B cell signaling.

### A module shared by B cells and monocytes; a gene expression program enabling antigen processing and presentation

While ***modules 22 and 38*** are specifics to B cells, ***module 41*** was strongly associated with both B cells and monocytes, two antigen-presenting cells (**Table 1**). Global analysis of the 39 genes within this module revealed multiple annotation terms (N = 66) that were significantly enriched, with the three most significant (%FDR<10^−8^) being “endosome”, “MHC classes I/II-like antigen recognition protein”, and “MHC class II protein complex” (**S4 Table**). In fact, many of the annotation terms found to be enriched in this module can be explained by a core set of genes including *CIITA*, *CD74*, *HLA-DMA*, *HLA-DRA*, *HLA-DRB1*, *HLA-DMB*, *CD1A*, *CD1C*, *LY86*, and *CD180*. The five TEGs in ***module 41*** are part of this core set of genes (*CIITA*, *CD74*, *HLA-DMA*, *HLA-DRA*, and *HLA-DRB1*), all involved in and essential to antigen presentation in B cells and monocytes [75, 76] (**S8 Fig**).

Regarding the genes that are most representative of ***module 41***, there were six MRGs (*CD74*, *SIDT2*, *FGD2*, *CD1A*, *MMP17*, and *HLA-DRA*), three of which are included in the core set of genes described above (*CD74*, *CD1A*, and *HLA-DRA*). Additionally, SIDT2 and FGD2 contribute to intracellular trafficking relevant to antigen processing [77, 78]. MMP17 is an endopeptidase that may be involved in the activation of membrane-bound precursors of growth factors or inflammatory mediators, such as TNF-α [79], which enhances antigen presentation. The combination of the global and targeted analyses clearly supports ***module 41*** having a role in antigen presentation in both B cells and monocytes.

### A shared module associated with NK, γδ T, and CD8+ T cells; a role in cytotoxic granule and non-self-recognition

**Fig 5:**
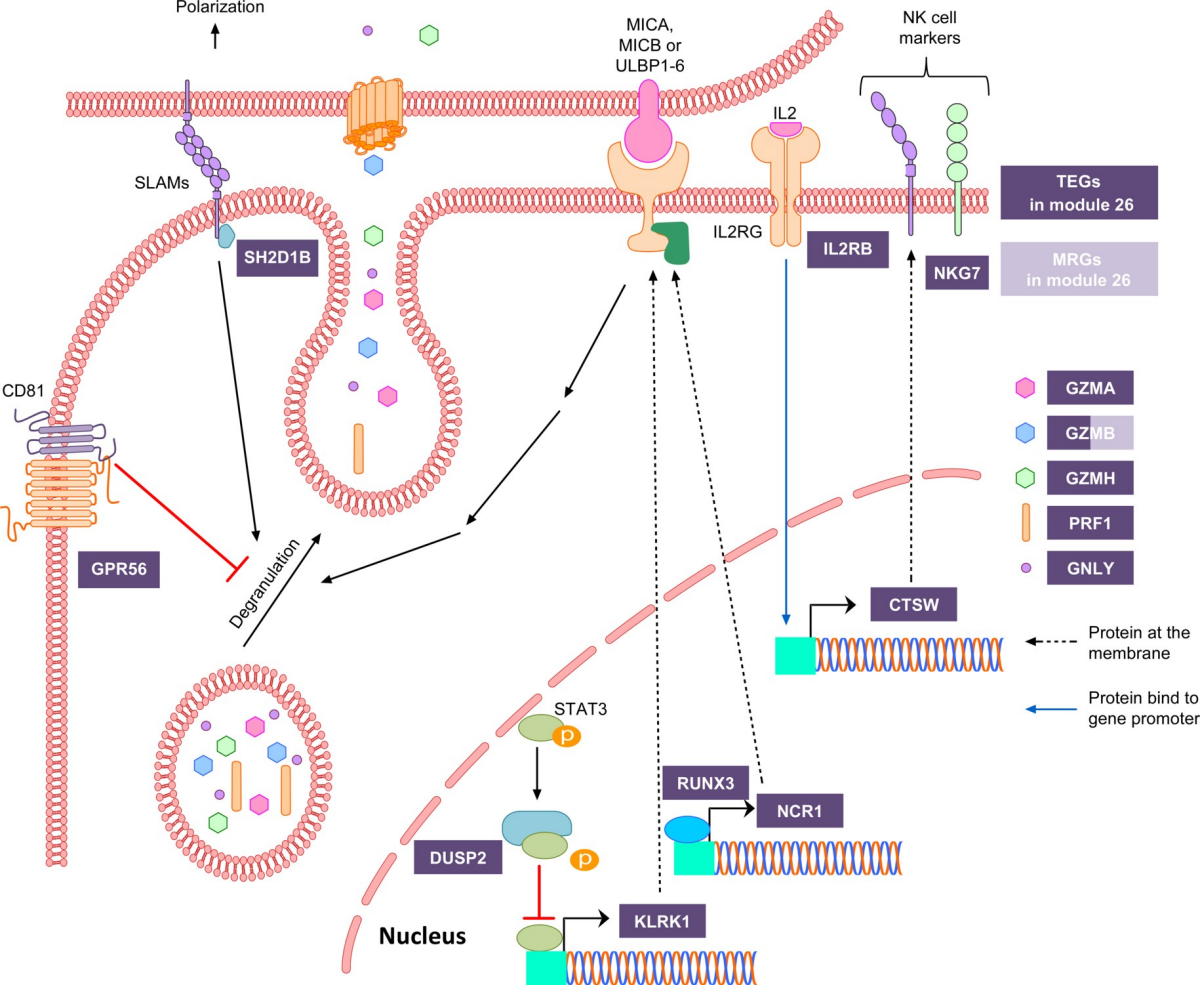
Targeted analyses of genes within module 26, associated with NK, γδ T, and CD8+ T cells. The majority of genes in ***module 26*** encode components of cytotoxic granules and proteins involved in the recognition of non-self. Genes in ***module 26*** are represented in purple: TEGs in dark purple and MRG in light purple. To see the profile of gene expression mean of all genes of module 26 presented in this Figure refers to the heatmap in S7 Fig.

**Fig 3**shows that ***module 26*** is associated with NK, γδ T and CD8+ T cells, strongly suggesting that it contains genes encoding for functions shared by these cell types (**Table 1**). The global analyses of the 129 genes within this module identified multiple significantly enriched annotation terms, including “natural killer cell-mediated cytotoxicity”, “cellular defense response” and “regulation of immune response”, all with FDR<10^−7^ (**S4 Table**). Genes enriched for these functions are located in the KIR locus (*KIR2DL1*, *KIR2DL3*, *KIR3DL1*, *KIR3DL2*, and *KIR2DS4*) within the human leukocyte receptor gene complex (LRC), in the NK gene complex (NKC) (*KLRC1*, *KLRC2*, *KLRC3*, *KLRC4*, *KLRD1*, *KLRF1*, and *KLRK1*), encoding proteins found in cytolytic granules (PRF1, GZMB, and GNLY) and cell surface proteins (*CD160*, *NCR1*, and *FASLG*) that mediate and/or potentiate cell-cell interaction. The primary functions that NK, γδ T, and CD8+ T cells have in common are the recognition and killing of abnormal cells (e.g. infected with viruses or other intracellular pathogens, cancerous), and the genes noted above are essential to these functions (**Fig 5**). It should be noted that all of the genes have equivalent expression levels across these three cell types, except for the KIR genes which had a differential expression pattern (NK > γδ T > CD8+ T).

When examining the 18 TEGs in these cell types, many of the same genes are highlighted (*GNLY*, *GZMB*, *KLRD1*, *KLRF1*, *KLRK1*, and *PRF1*), although other TEGs, such as *GZMA* and *GZMH*, can also be attributed to cytolytic granules. *NKG7* (alias *GMP-17*), another TEG, may also be contained within these granules in all three cell types, since it is found in the membrane of NK cell granules [80]. Many of the other TEGs within this module are involved in regulating the cell activation state, such as GPR56, an inhibitory receptor on NK cells [81], NCR1, that displays either a stimulatory or inhibitory effect depending on the ligand [82], and SH2D1B that regulates signal transduction from cell-surface SLAM receptors and leads to granule polarization to cell-cell synapses [83]. In addition, CD160 is potentially an activating receptor encoded outside the LRC and NKC complexes [84, 85].

Also included in this list of TEGs are a number of genes whose functions are poorly characterized in NK cells, γδ and CD8+ T cells, including *CST7*, *CTSW*, and *FGFBP2*. *CST7* encodes cystatin F, which may be important for the processing and activation of granule-associated serine proteases, in particular granzymes A and B [86]. *CTSW* encodes Cathepsin W, which is a putative cysteine protease that is believed to be released during target cell killing, although apparently not essential to the process of cytotoxicity [87]. *FGFBP2* is highly expressed in NK cells, γδ T and CD8+ T cells and essentially absent in the other immune cell types studied herein, suggesting that it plays a specific function in these cells.

Regarding the genes that are representative of ***module 26***, there were 21 MRGs, with three (*GZMB*, *KLRD1*, and *S1PR5*) also being in the list of TEGs (**S6 Table**). Five of the MRGs are encoded within the LRC or NKC complexes (*KLRD1*, *KIR3DL1*, *KIR2DL3*, *KLRC3*, and *KLRC1*). This MRG list also contained *FASLG*, important in T lymphocyte induced cell death, as well as *B3GAT1* which encodes a key enzyme in the biosynthesis of the carbohydrate epitope HNK-1 (human natural killer-1, alias CD57). Moreover, this list contains the genes that encode the XCL1 and XCL2 cytokines–two ligands for the chemokine receptor XCR1. Although still poorly characterized, this chemokine likely mediates the chemotactic activity of cDC towards cytotoxic cells [88].

In terms of transcriptional control of genes within this module, it is interesting to note that *DUSP2*, a TEG of this module, encodes a dual-specificity phosphatase that negatively regulates the activity of the signal transducer and transcriptional activator *STAT3*, which in turn regulates the transcription of the *KLRK1* gene within the NK gene complex [89, 90]. The TF RUNX3 is also a TEG and regulates genes implicated in lymphocyte activation, proliferation, cytotoxicity, migration and cytokine production in CD8+ T and NK cells, like *NCR1* and *IL2RB* [91, 92]. Although not within the list of TEGs, *ZNF683* (alias *Hobit*) drives the expression of *GPR56* in NK cells [81].

Taken together, these analyses suggest that ***module 26*** has two predominant functions, the first being cytotoxic granule composition, and the other being non-self-recognition, very consistent with the role of NK, γδ T, and CD8+ T cells.

### Modules associated with LPS-activated macrophages

In addition to identifying co-regulated genes associated with specific cell types, we also explored our ability to use this experimental approach to examine the transcriptional impact of modifying the activation state of a cell. Specifically, we cultured CD14+ enriched mononuclear cells from human peripheral blood with M-CSF for a total of 8 days to obtain macrophages [93, 94] and activated macrophages were generated by stimulating the macrophages with LPS during the last 24h of culture [95]. As can be seen in **Fig 3**, ***module 29*** stands out as being highly associated with response to LPS cells (**Table 1**). A global analysis of the 93 genes within this module identified 29 significantly enriched annotation terms (FDR<5%) that fell within two groups. The first group, including “cellular response to interferon-gamma”, “cellular response to interleukin-1”and “cellular response to tumor necrosis factor”, is driven by a common set of genes (*CCL1*, *ASS1*, *CCL20*, *EDN1*, *CCL8*, *CCL19*, *IRG1* (alias *ACOD1*), *CCL15* and *CCL17*), many of which are chemokines (*CCL* or *CXCL* genes) acting as chemoattractants for immune cells. *EDN1* is expressed by endothelial cells, although it can also be produced by macrophages co-cultured with activated T cells [96]. The second group includes annotations related to “metallothionein” and is also driven by a common set of genes (*MT1G*, *MT1H*, *MT1L*, *MT1M*, and *MT2A*) known as metallothioneins; they shape the macrophage zinc pool in response to inflammatory and infectious stimuli [97].

***Module 29***, strongly associated with LPS-activated macrophages, contains five TEGs; *CCL8* and *MT2A*, both highlighted by the global analysis, complement factor B, known to be LPS-induced [98], *IDO1*, which catalyzes the first and rate-limiting step of the catabolism of the essential amino acid tryptophan along the kynurenine pathway [99], and, finally, *ST3GAL1*, which is highly expressed in all cell types examined, and thus is likely, not informative to this module. In addition to the TEGs, 14 MRGs were found in ***module 29*** including three metallothioneins (*MT1G*, *MT1H*, and *MT1M*) and one chemokine (*CCL15*) also highlighted by the global analysis. Among the other MRGs, we found two cytokines, *CSF3* and *IL36G*, as well as *PDGFRL*, whose molecular function is not established but may be involved in cell proliferation [100]. Taken together, these analyses support an antimicrobial function for this module, with an important signature of chemokine production and zinc metabolism.

### The neutrophil transcriptome is distinctive from other immune cell types

Examining the distribution of reads, before and after normalization, we observed that neutrophils have a very different pattern when compared to the other cell types (**S2 Fig**). Neutrophils have fewer expressed genes (11,626 genes for neutrophil and between 13,161 and 13,614 for other cell populations) and the spread in expression is larger than the other cell types. Not surprisingly, this was also observed in the PCA and hierarchical clustering analyses, representing global differences in the transcriptome of neutrophils as compared to all other cell types examined herein (**Fig 1A**). In addition, there were 15 transcripts with a greater than the 100-fold difference between the average number of read counts in neutrophils as compared to the average number of read counts across all other cell types combined (**see S2 Text**).

Global analysis of the 13 neutrophil modules identified general functions such as “transcription” and “phosphoprotein”; however, a number of more specific functions relevant to neutrophils/myeloid cells were also identified, such as “lysosome”, “extracellular exosome”, “movement of cell or subcellular component”, and “superoxide-generating NADPH oxidase activator activity” (**S4 Table**). In terms of targeted analyses for these 13 neutrophil-associated modules, there were multiple TEGs (n = 349) and MRGs (n = 628). These analyses highlighted neutrophil polarity, lipid metabolism, and chemoattraction as functions related to ***module 4***; calprotectin, azurophil granules, cytoskeleton dynamics, chemoattraction for ***module 7***; actin remodeling & membrane trafficking, nicotinamide metabolism for ***module 10***; neutrophil polarization, trafficking and exocytosis for ***module 21***; and endocytosis and membrane trafficking for ***module 36***.

Moreover, given that the molecular aspects of many neutrophil functions have previously been characterized, we looked for the presence of the transcripts relevant to these functions within our modules. Specifically, we looked for 126 transcripts relevant to neutrophil granules (e.g. azurophil, gelatinase, secretory), antimicrobial proteins, reactive oxygen species (ROS), Neutrophil Extracellular Traps (NETs), cell cross-talk and resolution of inflammation (apoptosis and clearance, lipid mediator class switch) [101–104]. One hundred 11 transcripts were expressed in our dataset, with 80 of them found within our neutrophil-associated modules. Using this approach, we found that ***module 11*** was associated with the presence of transcripts for azurophil granules, ***module 15*** with defensin-specific granules, ***module 21*** with ROS and NET proteins, and ***modules 16*** with NET-associated Histone Cluster 1 family members. Extending this approach to the neutrophil modules also associated with other myeloid cells, we observe multiple transcripts relevant to neutrophil functions, either because the functions are shared between these cell types (e.g. ROS production, vesicle trafficking) or because the gene products are involved in different functions (e.g. actins are involved in NETs and other cytoskeleton-related functions).

### Transcriptional control of genes within B cell modules

In order to gain an understanding of the transcriptional control of genes within each module, we were interested in examining whether there was an enrichment of TF binding sites (TFBS) within the promoters of the genes within its module. In order to do so, we used the empirical ChIP-Seq data from the ENCODE (Encyclopedia of DNA Elements) Project [58]. Given that B lymphocytes are well represented in the ENCODE dataset—76 TFs were studied in the GM12878 immortalized B cell line–we focused our initial analyses on the three modules most associated with B lymphocytes and described above (***modules 22*, *38 and 41***). Specifically, these analyses identified that the TFBS for EZH2 was significantly enriched in the proximal promoters of the 195 genes within ***module 22*** and the TFBS for IKZF1 was enriched in the promoters of the 45 genes within ***module 38*** (**S7 Table**). Analysis of the promoters for genes within ***module 41*** associated with B cells and monocytes revealed enrichment for nine different TFBSs (BCL11A, CEBPB, EP300, EZH2, IKZF1, MTA3, NFATC1, RFX5, and SPI1) (**S7 Table**). Interestingly, the enrichment was the greatest for IKZF1, with over four- and five-fold enrichment in ***modules 38*, *and 41***, respectively. The transcript for IKZF1 is found in ***module 17***, which while associated with lymphoid cells (**S6 Fig**), has also strong expression across all immune cells (**S7 Table**). BCL11A is essential for lymphopoiesis and B cell development; it is also a critical component of a transcriptional network that regulates B cell fate by controlling V(D)J recombination and functions upstream of EBF1 and PAX5 in the B cell lineage differentiation pathway [105–107]. RFX5, EZH2, EP300, and SPI1 all control MHC class II expression either directly or via interaction or regulation of CIITA [108–115]. MTA3 has been implicated in cell fate during B lymphocyte differentiation [116]. NFATC1 regulates normal homeostasis and differentiation in B cells [117]. Finally, CEBPB is involved in the survival of Ly6C^-^ monocytes and have unknown function in MHC class II [118].

Interestingly, all of the TFs enriched in this analysis have average-to-high levels of expression in B cells. Among the 76 TFs that were evaluated, only two (MYC and NFATC1) have their TFBS enriched in their own modules (***modules 3 and 23***; respectively). Specifically, the empirical data provided evidence of a TFBS for MYC in the promoter region of 180 genes of the 619 genes from ***module 3***. It has been shown that MYC is necessary to stimulate both proliferation and inhibited differentiation in mature B cells induced by BCR signal [119, 120]. BCR signaling is also known to increase the transcription of the *NFACT1* gene, which in turn plays an important role in controlling plasmablast/plasma cell formation [121, 122].

### Transcription factors potentially associated with cell lineages and states

As described above for the analysis of the B cell-specific modules, the expression of TFs playing a key role in cell fate can be maintained in mature cells. In fact, with the exception of BACH2, TCF3 and IKZF1, all of the key TFs known to be involved in the multiple steps of B-cell-lineage differentiation from common lymphoid progenitor to mature B cell are within modules 22 and 38, despite their being identified from the transcriptome of mature B cells (**S8 Table**) [123–126]. *BACH2*, *TCF3*, and *IKZF1* were not identified in these analyses as, while they are necessary to B cell differentiation, their expression is not unique to B cells and in fact, they are ubiquitous in the primary immune cell types studied herein. We were therefore interested in identifying additional TFs that were highly expressed in a given cell type or cell lineage. In order to do so, we examined the 1600 known human TFs [44] for expression patterns that were consistent with the differentiation scheme depicted in **Fig 2B**. Specifically, we considered each branch point as representing a cell fate decision and looked for TFs that appeared to be specific for a given fate (e.g. myeloid vs. lymphoid; neutrophil vs. monocyte; B cell vs. T cell). For example, we looked for TFs that were highly expressed in lymphoid cells and absent or only very weakly in myeloid cells and identified 13 TFs that are candidates for being involved in lymphoid differentiation and/or function (**S5 Fig and S5 Table**). This list includes IKZF3, which is a known regulator of lymphoid development and BACH2, a known inhibitor of myeloid differentiation [127, 128]. In addition, when looking for TFs potentially involved in the differentiation step leading to monocytes, we identified SMAD1, which is known to be implicated in monocyte differentiation, polarization and inflammation [129, 130] and MITF which has not previously been associated with monocyte differentiation but is known to be a phagocyte-restricted TF in macrophage [131]. Altogether, we identified 74 TFs differentially expressed in one cell type or lineage (**S5 Fig and S5 Table**), with over a third (22/74) having confirmatory evidence in the literature as being involved in the relevant differentiation step. While not definitive proof, this certainly indicates that these TFs are strong candidates for playing a role in cell fate decisions and/or maintenance.

## Discussion

Cells within the immune system are generated through successive differentiation steps from a common pluripotent hematopoietic stem cell progenitor. As in all differentiation processes, certain functions are gained and others are lost along the way, such that each immune cell subset has different functions, some unique to a given cell type while others are shared. For decades, such differences have been exploited in flow cytometry-based experimentation, with specific cell surface markers (e.g. CD3, CD4, CD8, and CD14) used to identify, qualify and quantify immune cell populations within different experimental contexts. Sequencing the transcriptome (RNA-seq) of these immune cells can provide a complementary approach to understanding the differences between immune cell types or between different experimental conditions, disease states. RNA-seq based approaches certainly come with the challenge of analyzing and interpreting the data on thousands of transcripts per sample. We, therefore, set out to generate, analyze and interpret RNA-seq data of primary human immune cells from healthy individuals in order to evaluate the feasibility and usefulness to understand some of the biology underlying these cells and could play a major role in many immune-mediated diseases.

Starting with the premise that genes with correlated expression patterns are more likely to have shared biological functions than if they have disparate expression patterns, we aimed to identify functional modules within and across primary human immune cells by defining sets of co-expressed genes. Indeed, this unsupervised approach identified co-expression modules that were either highly correlated to a single cell type or to a group of cell types (**Fig 3**). We then assigned potential functions to these modules using a combination of global and targeted annotation approaches. From these analyses, it was clear that modules associated with a single cell type were linked to known functions of those cell types; for example BCR signaling pathway genes in the B cell-specific module or genes encoding proteins linked to functions of specific granules, ROS and NET production in different neutrophil-associated modules. As importantly, modules shared across different cell types also had biologic meaning; for example, MHC Class II antigen processing and presentation in B cells and monocytes, or cytotoxic granule composition and non-self-cell identification in NK, γδ T, and CD8+ T cells. These results confirm that this approach can reveal co-expressed genes that do share functions.

While not the primary objective of the current study, these co-expression modules also provide an opportunity to use a “guilt by association” approach in order to identify additional genes involved in the specific functions revealed by our analyses. For example, a number of genes in ***module 41*** encode proteins implicated in vesicular traffic (*BLOC1S6*, *LAMP5*, *TBC1D5*, and *TRAK1*), potentially revealing novel players in the trafficking of endosomal vesicles involved in antigen processing and presentation. Additional examples are: (1) two RAS family proteins, DIRAS1 and RASGRF1, that are potentially involved in B cell signaling [72]; (2) FGFBP2 potentially having a role in cytotoxic functions of NK, γδ T and CD8+ T cells [88]; (3) NECAB2 potentially being an important regulator of neutrophil apoptosis, autophagy and NETs; and (4) the enzyme-couple channel TRPM6 involved in neutrophil chemotaxis. While experimental validation is required to confirm these proposed functions, nonetheless, this illustrates how additional functional hypotheses can be generated from the information contained within the co-expression modules.

Also found within these modules are genes encoding TFs and transcriptional regulators; multiple TFs with known roles within the immune system were found within the relevant modules. Prime examples are the B cell-specific TFs PAX5 and EBF1 that are respectively within the B cell-specific ***modules 22 and 38***, CIITA is known to regulate MHC class II genes found within ***module 41***, and DUSP2, in ***module 26***, is a dual-specificity phosphatase that influences indirectly the expression of killer receptors within the NK gene complex. In order to assess whether TFs controlling the expression of genes within their own co-expression modules or in distinct modules, we focused our analyses on B cell modules, given the extensive ENCODE Chip-seq data for the 76 TFs in cell lines from the B cell lineage. While we detected an enrichment signal for 45 TFBS in 12 B cell-associated modules. Only two TFs (MYC and NFATC1) have their TFBS enriched in their own modules [119–122], supporting a network model of TFs regulating the transcription of genes in distinct co-expressed gene sets, which is consistent with current literature [19–21, 132].

While the focus of our analyses was to identify functional subgroups of genes, we did perform an analysis of TFs that were independent of the co-expression modules described above. Specifically, we searched for TFs that differentially expressed in a given cell type or lineage. Indeed, this approach identified many known lineage-specific TFs that are likely involved in maintaining lineage commitment and key functions associated with those commitments. Prime examples were IKZF3 known to be involved in lymphocyte development and homeostasis [127], PAX5 in differentiation and function of mature B cells [133], and FOXP3 in CD4+ T cell differentiation [134–136]. This analysis also highlighted a few TFs previously unknown to be associated with specific lineages, such as ZNF860 in B cell differentiation, ZNF385A in monocyte differentiation, CREB5 in the myeloid lineage, TFCP2L1 in γδ T lineage, and MSC in the activation of macrophages in response to LPS (**S5 Fig and S5 Table**). While the expression patterns were certainly consistent with a role in cell fate decision and/or maintenance, with many having published confirmatory evidence (18 of 49), future studies will be required to confirm the role of the remaining candidates.

In the future, the approach described herein could be used for the analysis of circulating immune cells in a variety of contexts, such as in cross-sectional comparisons between different immune-mediated diseases, in longitudinal studies of disease progression or response to therapy, or in a comparison of circulating immune cells versus immune cells taken from peripheral inflammatory sites. It is clear that comparisons in such study designs can be complex, with different expression patterns being a result of changes in cell populations, in addition to gene expression within cells, and therefore it will be important to rely on careful immunophenotyping and cell sorting strategies. Alternatively or in complementarity, with an inevitable decrease in the costs associated with single-cell RNA sequencing (scRNA-seq), this may become a better option, with the analysis strategy presented herein also being relevant to this experimental approach.

## Supporting information

S1 Text Analysis of all no-TEG and all no-MRG genes in *modules 22*, *38 and 41*. (DOCX)

S2 Text Additional analyses of genes highly-expressed in Neutrophils. (DOCX)

S1 Fig Immunophenotyping of immune cell populations from human peripheral blood. Results are expressed as median percentage among CD3+CD4+ (n = 12), CD3+CD8+ (n = 12) or CD3+ TCRγδ+ (n = 8) T lymphocyte populations respectively and among CD3-CD19+ B lymphocyte (n = 12), CD3-CD56+ NK cell (n = 12) or CD14+ monocyte (n = 12) populations, with quartiles [Q1-Q3] for each subset. CD14+ enriched mononuclear cells were stimulated *in vitro with* M-CSF in order to obtain macrophages, with an additional 24h LPS stimulation to obtain activated macrophages. Cells were stained with the mentioned surface markers and analyzed by flow cytometry. Results presented are the median MFI of specific staining minus unstained with quartiles [Q1-Q3]. [9, 95, 137–143].
(DOCX)

S2 Fig Boxplot of gene expression distribution after normalization before and after normalization. Each boxplot represents the mean of gene expression in log2 for a cell type in our 12 individuals. Panel A shows the log2 of cpm normalized by library size and TMM normalization factor. Panel B shows the distribution after variance stabilization by R function *voom*. Even after normalization with *voom*, a function of Limma package, the median of neutrophil gene distribution is not aligned with other cell types because many more genes are not expressed in neutrophils than other primary immune cell types in our data [29].
(DOCX)

S3 Fig Plot of scale independence and mean connectivity–Pearson correlation. Scale independence and mean value connectivity were used to select the β parameter, an exponent to the gene correlation matrix that determines the emphasis put on higher vs lower correlations [34]. Value 12 was chosen for β because of it as a good trade-off between scale-free topology (R^2 = 0.719) and connectivity. Therefore, β = 12 was used to compute dissimilarities between genes with the WGCNA functions, *adjacency* and *TomsimilarityFromExpr* (Topology Overlap Matrix Similarity From Expression) [33, 34]. The red line on scale independence graph represents value 0.8 (suggested by the authors).
(DOCX)

S4 Fig Hierarchical trees of gene modules before and after cut tree. Graph A represents the tree of modules obtained with WGCNA tools. The red line on this graph is the value 0.05 who chooses to cut the tree to grouping similar modules in one. Graph B represents the new modules after cuts tree with new numeration.
(DOCX)

S5 Fig Hematopoietic differentiation scheme and associated transcription factors from differential gene expression. To identify transcription factors consistent with having a role in cell fate decisions we examined differential gene expression for all known human transcription factors (n = 1638) [44]. Schematic simplification is used as a representation of hematopoiesis from lymphoid and myeloid lineage. Transcription factors are in red and black. Red represents transcription factors known to be involved in the establishment and/or maintaining cell/lineage differentiation. The pink background color is used for transcription factors associated with cytotoxic cells. Blue arrows show increased or decreased expression of genes coding for transcription factors. Complete list of candidate TFs in S5 Table
(DOCX)

S6 Fig Heatmap of the correlation values (and p-values) of WGCNA modules with primary immune cell types. Columns represent modules computed with WGCNA and rows, primary immune cell types. In each square, the first number represents the correlation between a module and a given cell type and the second number in brackets is the associated p-value.
(TIF)

S7 Fig Heatmap of mean normalized expression for a subset of genes. The heatmap represents gene normalized expression levels (log2 of cpm) in our nine cell types. Red is the higher value and yellow, the lower.
(DOCX)

S8 Fig Global and targeted analyses of genes within module 41, associated with B cells and monocytes, describe MHC class II and antigen processing and presentation functions. Global and targeted analyses of the genes within *module 41* were primarily associated with the presentation of peptide and lipid antigens. Genes in module 41 are represented in orange: *Top Expressing Genes* in dark orange, *Module Representative Genes* in intermediate orange and other genes in light orange. Genes from this module act together to establish Major Histocompatibility Complex class II function. To see the profile of gene expression mean of all genes of module 41 presented in this figure refers to the heatmap in S7 Fig.
(DOCX)

S1 Table List of antibodies used for immunophenotyping. (DOCX)

S2 Table List of antibodies used for monocyte/macrophage immunophenotyping. (DOCX)

S3 Table Summary statistics of RNA-Seq data from raw reads through quality control steps. Values are reads at each step.
(DOCX)

S4 Table Summary of gene annotation enrichments from DAVID tool (*P* ≤ 0.05). (XLSX)

S5 Table Differential gene expression and ratios of human transcription factors. First sheet: Differential gene expression and ratios of human TFs presented in S5 Fig. Second sheet: Differential gene expression and ratios of all known human TFs expressed in our immune cell dataset (n = 1112). Third sheet: List of all known human TFs not expressed in our immune cell dataset.
(XLSX)

S6 Table Percentile, mean, standard deviation, median, and IQR of gene expression read counts. First sheet: Mean of gene expression read count and percentile values. Second sheet: Standard deviation of gene expression read count. Third sheet: Median of gene expression read count. Fourth sheet: Interquartile range of gene expression read count.
(XLSX)

S7 Table Summary of transcription factor binding site or TFBS enrichments from the ENCODE project. Empirical ChIP-Seq data in the GM12878 immortalized B cell line was used within the promoters of the genes within each module associated with B lymphocytes (*P* ≤ 0.05).
(XLSX)

S8 Table Literature review of key transcription factors involved in B-cell differentiation and maturation. *** The TFs IRF4, PAX5, and BACH2, along with the absence of BCL6, have been reported to also play a role in the maturation of mature naïve to memory B cells. (26751566); Number in parenthesis is PMID number, which is the unique identifier number used in PubMed for each article.
(DOCX)

S1 Data (DOC)

## Abbreviations

TF: transcription factor
WGCNA: weighted gene co-expression network analysis
TEG: top expressing gene
MRG: module representative gene

## References

[1] Xiong T, Turner JE. Innate lymphoid cells in autoimmunity and chronic inflammatory diseases. Semin Immunopathol. 2018;40(4):393–406. Epub 2018/03/24. 10.1007/s00281-018-0670-4 . 29568972

[2] Pockley AG, Henderson B. Extracellular cell stress (heat shock) proteins-immune responses and disease: an overview. Philos Trans R Soc Lond B Biol Sci. 2018;373(1738). Epub 2017/12/06. 10.1098/rstb.2016.0522 29203707 PMC5717522

[3] Ribatti D. The concept of immune surveillance against tumors. The first theories. Oncotarget. 2017;8(4):7175–80. Epub 2016/10/21. 10.18632/oncotarget.12739 27764780 PMC5351698

[4] Conway EM, Pikor LA, Kung SH, Hamilton MJ, Lam S, Lam WL, et al Macrophages, Inflammation, and Lung Cancer. Am J Respir Crit Care Med. 2016;193(2):116–30. Epub 2015/11/20. 10.1164/rccm.201508-1545CI . 26583808

[5] Davis MM, Tato CM, Furman D. Systems immunology: just getting started. Nat Immunol. 2017;18(7):725–32. Epub 2017/06/21. 10.1038/ni.3768 28632713 PMC5790187

[6] Abbas AK, Lichtman AH, Pillai S. Cellular and Molecular Immunology. edition t, editor2017 11th May 2017.

[7] Murphy KM, Weaver C. Janeway's Immunobiology. edition t, editor2016. 904 p.

[8] Underhill DM, Gordon S, Imhof BA, Nunez G, Bousso P. Elie Metchnikoff (1845–1916): celebrating 100 years of cellular immunology and beyond. Nat Rev Immunol. 2016;16(10):651–6. Epub 2016/08/02. 10.1038/nri.2016.89 . 27477126

[9] Maecker HT, McCoy JP, Nussenblatt R. Standardizing immunophenotyping for the Human Immunology Project. Nat Rev Immunol. 2012;12(3):191–200. Epub 2012/02/22. 10.1038/nri3158 22343568 PMC3409649

[10] Golub TR, Slonim DK, Tamayo P, Huard C, Gaasenbeek M, Mesirov JP, et al Molecular classification of cancer: class discovery and class prediction by gene expression monitoring. Science. 1999;286(5439):531–7. Epub 1999/10/16. 10.1126/science.286.5439.531 . 10521349

[11] Bennett L, Palucka AK, Arce E, Cantrell V, Borvak J, Banchereau J, et al Interferon and granulopoiesis signatures in systemic lupus erythematosus blood. J Exp Med. 2003;197(6):711–23. Epub 2003/03/19. 10.1084/jem.20021553 12642603 PMC2193846

[12] Lowes MA, Suarez-Farinas M, Krueger JG. Immunology of psoriasis. Annu Rev Immunol. 2014;32:227–55. Epub 2014/03/25. 10.1146/annurev-immunol-032713-120225 24655295 PMC4229247

[13] Kim CC, Lanier LL. Beyond the transcriptome: completion of act one of the Immunological Genome Project. Curr Opin Immunol. 2013;25(5):593–7. Epub 2013/10/31. 10.1016/j.coi.2013.09.013 24168965 PMC3855358

[14] Heng TS, Painter MW, Immunological Genome Project C. The Immunological Genome Project: networks of gene expression in immune cells. Nat Immunol. 2008;9(10):1091–4. Epub 2008/09/19. 10.1038/ni1008-1091 . 18800157

[15] Proserpio V, Mahata B. Single-cell technologies to study the immune system. Immunology. 2016;147(2):133–40. Epub 2015/11/10. 10.1111/imm.12553 26551575 PMC4717243

[16] Shay T, Kang J. Immunological Genome Project and systems immunology. Trends Immunol. 2013;34(12):602–9. Epub 2013/05/02. 10.1016/j.it.2013.03.004 23631936 PMC4615706

[17] Bjorklund AK, Forkel M, Picelli S, Konya V, Theorell J, Friberg D, et al The heterogeneity of human CD127(+) innate lymphoid cells revealed by single-cell RNA sequencing. Nat Immunol. 2016;17(4):451–60. Epub 2016/02/16. 10.1038/ni.3368 . 26878113

[18] Villani AC, Satija R, Reynolds G, Sarkizova S, Shekhar K, Fletcher J, et al Single-cell RNA-seq reveals new types of human blood dendritic cells, monocytes, and progenitors. Science. 2017;356(6335). Epub 2017/04/22. 10.1126/science.aah4573 28428369 PMC5775029

[19] Mason MJ, Fan G, Plath K, Zhou Q, Horvath S. Signed weighted gene co-expression network analysis of transcriptional regulation in murine embryonic stem cells. BMC Genomics. 2009;10:327 Epub 2009/07/22. 10.1186/1471-2164-10-327 19619308 PMC2727539

[20] Mabbott NA, Baillie JK, Brown H, Freeman TC, Hume DA. An expression atlas of human primary cells: inference of gene function from coexpression networks. BMC Genomics. 2013;14:632 Epub 2013/09/24. 10.1186/1471-2164-14-632 24053356 PMC3849585

[21] van Dam S, Vosa U, van der Graaf A, Franke L, de Magalhaes JP. Gene co-expression analysis for functional classification and gene-disease predictions. Brief Bioinform. 2018;19(4):575–92. Epub 2017/01/13. 10.1093/bib/bbw139 28077403 PMC6054162

[22] Bolger AM, Lohse M, Usadel B. Trimmomatic: a flexible trimmer for Illumina sequence data. Bioinformatics. 2014;30(15):2114–20. Epub 2014/04/04. 10.1093/bioinformatics/btu170 24695404 PMC4103590

[23] Kim D, Pertea G, Trapnell C, Pimentel H, Kelley R, Salzberg SL. TopHat2: accurate alignment of transcriptomes in the presence of insertions, deletions and gene fusions. Genome Biol. 2013;14(4):R36 Epub 2013/04/27. 10.1186/gb-2013-14-4-r36 23618408 PMC4053844

[24] Li H, Handsaker B, Wysoker A, Fennell T, Ruan J, Homer N, et al The Sequence Alignment/Map format and SAMtools. Bioinformatics. 2009;25(16):2078–9. Epub 2009/06/10. 10.1093/bioinformatics/btp352 19505943 PMC2723002

[25] Delhomme N, Padioleau I, Furlong EE, Steinmetz LM. easyRNASeq: a bioconductor package for processing RNA-Seq data. Bioinformatics. 2012;28(19):2532–3. Epub 2012/08/01. 10.1093/bioinformatics/bts477 22847932 PMC3463124

[26] Robinson MD, McCarthy DJ, Smyth GK. edgeR: a Bioconductor package for differential expression analysis of digital gene expression data. Bioinformatics. 2010;26(1):139–40. Epub 2009/11/17. 10.1093/bioinformatics/btp616 19910308 PMC2796818

[27] McCarthy DJ, Chen Y, Smyth GK. Differential expression analysis of multifactor RNA-Seq experiments with respect to biological variation. Nucleic Acids Res. 2012;40(10):4288–97. Epub 2012/01/31. 10.1093/nar/gks042 22287627 PMC3378882

[28] Ritchie ME, Phipson B, Wu D, Hu Y, Law CW, Shi W, et al limma powers differential expression analyses for RNA-sequencing and microarray studies. Nucleic Acids Res. 2015;43(7):e47 Epub 2015/01/22. 10.1093/nar/gkv007 25605792 PMC4402510

[29] Law CW, Chen Y, Shi W, Smyth GK. voom: Precision weights unlock linear model analysis tools for RNA-seq read counts. Genome Biol. 2014;15(2):R29 Epub 2014/02/04. 10.1186/gb-2014-15-2-r29 24485249 PMC4053721

[30] Joliffe IT, Morgan BJ. Principal component analysis and exploratory factor analysis. Stat Methods Med Res. 1992;1(1):69–95. Epub 1992/01/01. 10.1177/096228029200100105 . 1341653

[31] Jolliffe IT, Cadima J. Principal component analysis: a review and recent developments. Philos Trans A Math Phys Eng Sci. 2016;374(2065):20150202 Epub 2016/03/10. 10.1098/rsta.2015.0202 26953178 PMC4792409

[32] Sainani KL. Introduction to principal components analysis. PM R. 2014;6(3):275–8. Epub 2014/02/26. 10.1016/j.pmrj.2014.02.001 . 24565515

[33] Langfelder P, Horvath S. WGCNA: an R package for weighted correlation network analysis. BMC Bioinformatics. 2008;9:559 Epub 2008/12/31. 10.1186/1471-2105-9-559 19114008 PMC2631488

[34] Zhang B, Horvath S. A general framework for weighted gene co-expression network analysis. Stat Appl Genet Mol Biol. 2005;4:Article17. Epub 2006/05/02. 10.2202/1544-6115.1128 . 16646834

[35] Langfelder P, Zhang B, Horvath S. Defining clusters from a hierarchical cluster tree: the Dynamic Tree Cut package for R. Bioinformatics. 2008;24(5):719–20. Epub 2007/11/21. 10.1093/bioinformatics/btm563 . 18024473

[36] Huang da W, Sherman BT, Lempicki RA. Systematic and integrative analysis of large gene lists using DAVID bioinformatics resources. Nat Protoc. 2009;4(1):44–57. Epub 2009/01/10. 10.1038/nprot.2008.211 . 19131956

[37] Bairoch A, Apweiler R, Wu CH, Barker WC, Boeckmann B, Ferro S, et al The Universal Protein Resource (UniProt). Nucleic Acids Res. 2005;33(Database issue):D154–9. Epub 2004/12/21. 10.1093/nar/gki070 15608167 PMC540024

[38] Mitchell A, Chang HY, Daugherty L, Fraser M, Hunter S, Lopez R, et al The InterPro protein families database: the classification resource after 15 years. Nucleic Acids Res. 2015;43(Database issue):D213–21. Epub 2014/11/28. 10.1093/nar/gku1243 25428371 PMC4383996

[39] Gene Ontology C. Gene Ontology Consortium: going forward. Nucleic Acids Res. 2015;43(Database issue):D1049–56. Epub 2014/11/28. 10.1093/nar/gku1179 25428369 PMC4383973

[40] Kanehisa M, Sato Y, Kawashima M, Furumichi M, Tanabe M. KEGG as a reference resource for gene and protein annotation. Nucleic Acids Res. 2016;44(D1):D457–62. Epub 2015/10/18. 10.1093/nar/gkv1070 26476454 PMC4702792

[41] Safran M, Dalah I, Alexander J, Rosen N, Iny Stein T, Shmoish M, et al GeneCards Version 3: the human gene integrator. Database (Oxford). 2010;2010:baq020 Epub 2010/08/07. 10.1093/database/baq020 20689021 PMC2938269

[42] Lu Z. PubMed and beyond: a survey of web tools for searching biomedical literature. Database (Oxford). 2011;2011:baq036 Epub 2011/01/20. 10.1093/database/baq036 21245076 PMC3025693

[43] Anders ME, Evans DP. Comparison of PubMed and Google Scholar literature searches. Respir Care. 2010;55(5):578–83. Epub 2010/04/28. . 20420728

[44] Lambert SA, Jolma A, Campitelli LF, Das PK, Yin Y, Albu M, et al The Human Transcription Factors. Cell. 2018;172(4):650–65. Epub 2018/02/10. 10.1016/j.cell.2018.01.029 . 29425488 PMC12908702

[45] Matys V, Kel-Margoulis OV, Fricke E, Liebich I, Land S, Barre-Dirrie A, et al TRANSFAC and its module TRANSCompel: transcriptional gene regulation in eukaryotes. Nucleic Acids Res. 2006;34(Database issue):D108–10. Epub 2005/12/31. 10.1093/nar/gkj143 16381825 PMC1347505

[46] Mathelier A, Fornes O, Arenillas DJ, Chen CY, Denay G, Lee J, et al JASPAR 2016: a major expansion and update of the open-access database of transcription factor binding profiles. Nucleic Acids Res. 2016;44(D1):D110–5. Epub 2015/11/05. 10.1093/nar/gkv1176 26531826 PMC4702842

[47] Jolma A, Yan J, Whitington T, Toivonen J, Nitta KR, Rastas P, et al DNA-binding specificities of human transcription factors. Cell. 2013;152(1–2):327–39. Epub 2013/01/22. 10.1016/j.cell.2012.12.009 . 23332764

[48] Jolma A, Yin Y, Nitta KR, Dave K, Popov A, Taipale M, et al DNA-dependent formation of transcription factor pairs alters their binding specificity. Nature. 2015;527(7578):384–8. Epub 2015/11/10. 10.1038/nature15518 . 26550823

[49] Yin Y, Morgunova E, Jolma A, Kaasinen E, Sahu B, Khund-Sayeed S, et al Impact of cytosine methylation on DNA binding specificities of human transcription factors. Science. 2017;356(6337). Epub 2017/05/06. 10.1126/science.aaj2239 . 28473536 PMC8009048

[50] Hume MA, Barrera LA, Gisselbrecht SS, Bulyk ML. UniPROBE, update 2015: new tools and content for the online database of protein-binding microarray data on protein-DNA interactions. Nucleic Acids Res. 2015;43(Database issue):D117–22. Epub 2014/11/08. 10.1093/nar/gku1045 25378322 PMC4383892

[51] Weirauch MT, Yang A, Albu M, Cote AG, Montenegro-Montero A, Drewe P, et al Determination and inference of eukaryotic transcription factor sequence specificity. Cell. 2014;158(6):1431–43. Epub 2014/09/13. 10.1016/j.cell.2014.08.009 25215497 PMC4163041

[52] Fulton DL, Sundararajan S, Badis G, Hughes TR, Wasserman WW, Roach JC, et al TFCat: the curated catalog of mouse and human transcription factors. Genome Biol. 2009;10(3):R29 Epub 2009/03/17. 10.1186/gb-2009-10-3-r29 19284633 PMC2691000

[53] Vaquerizas JM, Kummerfeld SK, Teichmann SA, Luscombe NM. A census of human transcription factors: function, expression and evolution. Nat Rev Genet. 2009;10(4):252–63. Epub 2009/03/11. 10.1038/nrg2538 . 19274049

[54] Wingender E, Schoeps T, Haubrock M, Donitz J. TFClass: a classification of human transcription factors and their rodent orthologs. Nucleic Acids Res. 2015;43(Database issue):D97–102. Epub 2014/11/02. 10.1093/nar/gku1064 25361979 PMC4383905

[55] Kinsella RJ, Kahari A, Haider S, Zamora J, Proctor G, Spudich G, et al Ensembl BioMarts: a hub for data retrieval across taxonomic space. Database (Oxford). 2011;2011:bar030 Epub 2011/07/26. 10.1093/database/bar030 21785142 PMC3170168

[56] Tompa M, Li N, Bailey TL, Church GM, De Moor B, Eskin E, et al Assessing computational tools for the discovery of transcription factor binding sites. Nat Biotechnol. 2005;23(1):137–44. Epub 2005/01/08. 10.1038/nbt1053 . 15637633

[57] Veerla S, Ringner M, Hoglund M. Genome-wide transcription factor binding site/promoter databases for the analysis of gene sets and co-occurrence of transcription factor binding motifs. BMC Genomics. 2010;11:145 Epub 2010/03/03. 10.1186/1471-2164-11-145 20193056 PMC2841680

[58] Consortium EP. An integrated encyclopedia of DNA elements in the human genome. Nature. 2012;489(7414):57–74. Epub 2012/09/08. 10.1038/nature11247 22955616 PMC3439153

[59] Kuhn RM, Haussler D, Kent WJ. The UCSC genome browser and associated tools. Brief Bioinform. 2013;14(2):144–61. Epub 2012/08/22. 10.1093/bib/bbs038 22908213 PMC3603215

[60] Monaco G, Lee B, Xu W, Mustafah S, Hwang YY, Carre C, et al RNA-Seq Signatures Normalized by mRNA Abundance Allow Absolute Deconvolution of Human Immune Cell Types. Cell Rep. 2019;26(6):1627–40 e7. Epub 2019/02/07. 10.1016/j.celrep.2019.01.041 30726743 PMC6367568

[61] Mootha VK, Lepage P, Miller K, Bunkenborg J, Reich M, Hjerrild M, et al Identification of a gene causing human cytochrome c oxidase deficiency by integrative genomics. Proc Natl Acad Sci U S A. 2003;100(2):605–10. Epub 2003/01/17. 10.1073/pnas.242716699 12529507 PMC141043

[62] Dylke J, Lopes J, Dang-Lawson M, Machtaler S, Matsuuchi L. Role of the extracellular and transmembrane domain of Ig-alpha/beta in assembly of the B cell antigen receptor (BCR). Immunol Lett. 2007;112(1):47–57. Epub 2007/08/07. 10.1016/j.imlet.2007.06.005 . 17675166

[63] Nutt SL, Kee BL. The transcriptional regulation of B cell lineage commitment. Immunity. 2007;26(6):715–25. Epub 2007/06/22. 10.1016/j.immuni.2007.05.010 . 17582344

[64] Cobaleda C, Schebesta A, Delogu A, Busslinger M. Pax5: the guardian of B cell identity and function. Nat Immunol. 2007;8(5):463–70. Epub 2007/04/19. 10.1038/ni1454 . 17440452

[65] Zandi S, Mansson R, Tsapogas P, Zetterblad J, Bryder D, Sigvardsson M. EBF1 is essential for B-lineage priming and establishment of a transcription factor network in common lymphoid progenitors. J Immunol. 2008;181(5):3364–72. Epub 2008/08/21. 10.4049/jimmunol.181.5.3364 . 18714008

[66] Blackburn TE, Santiago T, Burrows PD. FCRLA-A Resident Endoplasmic Reticulum Protein that Associates with Multiple Immunoglobulin Isotypes in B Lineage Cells. Curr Top Microbiol Immunol. 2017;408:47–65. Epub 2017/09/08. 10.1007/82_2017_40 . 28879521

[67] Muller J, Nitschke L. The role of CD22 and Siglec-G in B-cell tolerance and autoimmune disease. Nat Rev Rheumatol. 2014;10(7):422–8. Epub 2014/04/26. 10.1038/nrrheum.2014.54 . 24763061

[68] Rinaldi A, Chiaravalli AM, Mian M, Zucca E, Tibiletti MG, Capella C, et al Serotonin receptor 3A expression in normal and neoplastic B cells. Pathobiology. 2010;77(3):129–35. Epub 2010/06/03. 10.1159/000292646 . 20516728

[69] Li S, Liu J, Min Q, Ikawa T, Yasuda S, Yang Y, et al Kelch-like protein 14 promotes B-1a but suppresses B-1b cell development. Int Immunol. 2018;30(7):311–8. Epub 2018/06/26. 10.1093/intimm/dxy033 . 29939266

[70] Hagiwara S. Transgenic expression of VpreB-3 under the control of the immunoglobulin heavy chain enhancer and SV40 promoter. Kobe J Med Sci. 1996;42(1):43–59. Epub 1996/02/01. . 8984229

[71] Ebeling O, Duczmal A, Aigner S, Geiger C, Schollhammer S, Kemshead JT, et al L1 adhesion molecule on human lymphocytes and monocytes: expression and involvement in binding to alpha v beta 3 integrin. Eur J Immunol. 1996;26(10):2508–16. Epub 1996/10/01. 10.1002/eji.1830261035 . 8898967

[72] Tabrizi SJ, Niiro H, Masui M, Yoshimoto G, Iino T, Kikushige Y, et al T cell leukemia/lymphoma 1 and galectin-1 regulate survival/cell death pathways in human naive and IgM+ memory B cells through altering balances in Bcl-2 family proteins. J Immunol. 2009;182(3):1490–9. Epub 2009/01/22. 10.4049/jimmunol.182.3.1490 . 19155496

[73] Imamura Y, Oda A, Katahira T, Bundo K, Pike KA, Ratcliffe MJ, et al BLNK binds active H-Ras to promote B cell receptor-mediated capping and ERK activation. J Biol Chem. 2009;284(15):9804–13. Epub 2009/02/17. 10.1074/jbc.M809051200 19218240 PMC2665102

[74] Kabak S, Skaggs BJ, Gold MR, Affolter M, West KL, Foster MS, et al The direct recruitment of BLNK to immunoglobulin alpha couples the B-cell antigen receptor to distal signaling pathways. Mol Cell Biol. 2002;22(8):2524–35. Epub 2002/03/23. 10.1128/MCB.22.8.2524-2535.2002 11909947 PMC133735

[75] Klein J, Sato A. The HLA system. Second of two parts. N Engl J Med. 2000;343(11):782–6. Epub 2000/09/14. 10.1056/NEJM200009143431106 . 10984567

[76] Fortin JS, Cloutier M, Thibodeau J. Exposing the Specific Roles of the Invariant Chain Isoforms in Shaping the MHC Class II Peptidome. Front Immunol. 2013;4:443 Epub 2014/01/01. 10.3389/fimmu.2013.00443 24379812 PMC3861868

[77] Nguyen TA, Smith BRC, Tate MD, Belz GT, Barrios MH, Elgass KD, et al SIDT2 Transports Extracellular dsRNA into the Cytoplasm for Innate Immune Recognition. Immunity. 2017;47(3):498–509 e6. Epub 2017/09/17. 10.1016/j.immuni.2017.08.007 28916264 PMC5679266

[78] Huber C, Martensson A, Bokoch GM, Nemazee D, Gavin AL. FGD2, a CDC42-specific exchange factor expressed by antigen-presenting cells, localizes to early endosomes and active membrane ruffles. J Biol Chem. 2008;283(49):34002–12. Epub 2008/10/08. 10.1074/jbc.M803957200 18838382 PMC2590680

[79] English WR, Puente XS, Freije JM, Knauper V, Amour A, Merryweather A, et al Membrane type 4 matrix metalloproteinase (MMP17) has tumor necrosis factor-alpha convertase activity but does not activate pro-MMP2. J Biol Chem. 2000;275(19):14046–55. Epub 2000/05/09. 10.1074/jbc.275.19.14046 . 10799478

[80] Medley QG, Kedersha N, O'Brien S, Tian Q, Schlossman SF, Streuli M, et al Characterization of GMP-17, a granule membrane protein that moves to the plasma membrane of natural killer cells following target cell recognition. Proc Natl Acad Sci U S A. 1996;93(2):685–9. Epub 1996/01/23. 10.1073/pnas.93.2.685 8570616 PMC40113

[81] Chang GW, Hsiao CC, Peng YM, Vieira Braga FA, Kragten NA, Remmerswaal EB, et al The Adhesion G Protein-Coupled Receptor GPR56/ADGRG1 Is an Inhibitory Receptor on Human NK Cells. Cell Rep. 2016;15(8):1757–70. Epub 2016/05/18. 10.1016/j.celrep.2016.04.053 . 27184850

[82] Pazina T, Shemesh A, Brusilovsky M, Porgador A, Campbell KS. Regulation of the Functions of Natural Cytotoxicity Receptors by Interactions with Diverse Ligands and Alterations in Splice Variant Expression. Front Immunol. 2017;8:369 Epub 2017/04/21. 10.3389/fimmu.2017.00369 28424697 PMC5371597

[83] Perez-Quintero LA, Roncagalli R, Guo H, Latour S, Davidson D, Veillette A. EAT-2, a SAP-like adaptor, controls NK cell activation through phospholipase Cgamma, Ca++, and Erk, leading to granule polarization. J Exp Med. 2014;211(4):727–42. Epub 2014/04/02. 10.1084/jem.20132038 24687958 PMC3978279

[84] Le Bouteiller P, Tabiasco J, Polgar B, Kozma N, Giustiniani J, Siewiera J, et al CD160: a unique activating NK cell receptor. Immunol Lett. 2011;138(2):93–6. Epub 2011/02/18. 10.1016/j.imlet.2011.02.003 . 21324341

[85] Cai G, Freeman GJ. The CD160, BTLA, LIGHT/HVEM pathway: a bidirectional switch regulating T-cell activation. Immunol Rev. 2009;229(1):244–58. Epub 2009/05/12. 10.1111/j.1600-065X.2009.00783.x . 19426226

[86] Maher K, Konjar S, Watts C, Turk B, Kopitar-Jerala N. Cystatin F regulates proteinase activity in IL-2-activated natural killer cells. Protein Pept Lett. 2014;21(9):957–65. Epub 2014/04/08. 10.2174/0929866521666140403124146 . 24702263

[87] Stoeckle C, Gouttefangeas C, Hammer M, Weber E, Melms A, Tolosa E. Cathepsin W expressed exclusively in CD8+ T cells and NK cells, is secreted during target cell killing but is not essential for cytotoxicity in human CTLs. Exp Hematol. 2009;37(2):266–75. Epub 2008/12/23. 10.1016/j.exphem.2008.10.011 . 19100676

[88] Yamazaki C, Miyamoto R, Hoshino K, Fukuda Y, Sasaki I, Saito M, et al Conservation of a chemokine system, XCR1 and its ligand, XCL1, between human and mice. Biochem Biophys Res Commun. 2010;397(4):756–61. Epub 2010/06/15. 10.1016/j.bbrc.2010.06.029 . 20541533

[89] Lu D, Liu L, Ji X, Gao Y, Chen X, Liu Y, et al The phosphatase DUSP2 controls the activity of the transcription activator STAT3 and regulates TH17 differentiation. Nat Immunol. 2015;16(12):1263–73. Epub 2015/10/20. 10.1038/ni.3278 . 26479789

[90] Zhu S, Phatarpekar PV, Denman CJ, Senyukov VV, Somanchi SS, Nguyen-Jackson HT, et al Transcription of the activating receptor NKG2D in natural killer cells is regulated by STAT3 tyrosine phosphorylation. Blood. 2014;124(3):403–11. Epub 2014/06/04. 10.1182/blood-2013-05-499707 24891320 PMC4102712

[91] Lotem J, Levanon D, Negreanu V, Leshkowitz D, Friedlander G, Groner Y. Runx3-mediated transcriptional program in cytotoxic lymphocytes. PLoS One. 2013;8(11):e80467 Epub 2013/11/16. 10.1371/journal.pone.0080467 24236182 PMC3827420

[92] Ohno S, Sato T, Kohu K, Takeda K, Okumura K, Satake M, et al Runx proteins are involved in regulation of CD122, Ly49 family and IFN-gamma expression during NK cell differentiation. Int Immunol. 2008;20(1):71–9. Epub 2007/11/16. 10.1093/intimm/dxm120 . 18003603

[93] Picozza M, Battistini L, Borsellino G. Mononuclear phagocytes and marker modulation: when CD16 disappears, CD38 takes the stage. Blood. 2013;122(3):456–7. Epub 2013/07/23. 10.1182/blood-2013-05-500058 . 23869076

[94] Sierra-Filardi E, Nieto C, Dominguez-Soto A, Barroso R, Sanchez-Mateos P, Puig-Kroger A, et al CCL2 shapes macrophage polarization by GM-CSF and M-CSF: identification of CCL2/CCR2-dependent gene expression profile. J Immunol. 2014;192(8):3858–67. Epub 2014/03/19. 10.4049/jimmunol.1302821 . 24639350

[95] Verreck FA, de Boer T, Langenberg DM, van der Zanden L, Ottenhoff TH. Phenotypic and functional profiling of human proinflammatory type-1 and anti-inflammatory type-2 macrophages in response to microbial antigens and IFN-gamma- and CD40L-mediated costimulation. J Leukoc Biol. 2006;79(2):285–93. Epub 2005/12/07. 10.1189/jlb.0105015 . 16330536

[96] Shinagawa S, Okazaki T, Ikeda M, Yudoh K, Kisanuki YY, Yanagisawa M, et al T cells upon activation promote endothelin 1 production in monocytes via IFN-gamma and TNF-alpha. Sci Rep. 2017;7(1):14500 Epub 2017/11/05. 10.1038/s41598-017-14202-5 29101349 PMC5670167

[97] Subramanian Vignesh K, Deepe GS Jr. Metallothioneins: Emerging Modulators in Immunity and Infection. Int J Mol Sci. 2017;18(10). Epub 2017/10/27. 10.3390/ijms18102197 29065550 PMC5666878

[98] Huang Y, Krein PM, Muruve DA, Winston BW. Complement factor B gene regulation: synergistic effects of TNF-alpha and IFN-gamma in macrophages. J Immunol. 2002;169(5):2627–35. Epub 2002/08/24. 10.4049/jimmunol.169.5.2627 . 12193734

[99] Metz R, Duhadaway JB, Kamasani U, Laury-Kleintop L, Muller AJ, Prendergast GC. Novel tryptophan catabolic enzyme IDO2 is the preferred biochemical target of the antitumor indoleamine 2,3-dioxygenase inhibitory compound D-1-methyl-tryptophan. Cancer Res. 2007;67(15):7082–7. Epub 2007/08/03. 10.1158/0008-5472.CAN-07-1872 . 17671174

[100] Kawata K, Kubota S, Eguchi T, Aoyama E, Moritani NH, Oka M, et al A Tumor Suppressor Gene Product, Platelet-Derived Growth Factor Receptor-Like Protein Controls Chondrocyte Proliferation and Differentiation. J Cell Biochem. 2017;118(11):4033–44. Epub 2017/04/14. 10.1002/jcb.26059 . 28407304

[101] Amulic B, Cazalet C, Hayes GL, Metzler KD, Zychlinsky A. Neutrophil function: from mechanisms to disease. Annu Rev Immunol. 2012;30:459–89. Epub 2012/01/10. 10.1146/annurev-immunol-020711-074942 . 22224774

[102] Cowland JB, Borregaard N. Granulopoiesis and granules of human neutrophils. Immunol Rev. 2016;273(1):11–28. Epub 2016/08/26. 10.1111/imr.12440 . 27558325

[103] Jaillon S, Peri G, Delneste Y, Fremaux I, Doni A, Moalli F, et al The humoral pattern recognition receptor PTX3 is stored in neutrophil granules and localizes in extracellular traps. J Exp Med. 2007;204(4):793–804. Epub 2007/03/29. 10.1084/jem.20061301 17389238 PMC2118544

[104] Vorobjeva NV, Pinegin BV. Neutrophil extracellular traps: mechanisms of formation and role in health and disease. Biochemistry (Mosc). 2014;79(12):1286–96. Epub 2015/02/27. 10.1134/S0006297914120025 . 25716722

[105] Yu Y, Wang J, Khaled W, Burke S, Li P, Chen X, et al Bcl11a is essential for lymphoid development and negatively regulates p53. J Exp Med. 2012;209(13):2467–83. Epub 2012/12/12. 10.1084/jem.20121846 23230003 PMC3526365

[106] Liu P, Keller JR, Ortiz M, Tessarollo L, Rachel RA, Nakamura T, et al Bcl11a is essential for normal lymphoid development. Nat Immunol. 2003;4(6):525–32. Epub 2003/04/30. 10.1038/ni925 . 12717432

[107] Lee BS, Lee BK, Iyer VR, Sleckman BP, Shaffer AL 3rd, Ippolito GC, et al Corrected and Republished from: BCL11A Is a Critical Component of a Transcriptional Network That Activates RAG Expression and V(D)J Recombination. Mol Cell Biol. 2018;38(1). Epub 2017/10/19. 10.1128/MCB.00362-17 29038163 PMC5730723

[108] Garvie CW, Stagno JR, Reid S, Singh A, Harrington E, Boss JM. Characterization of the RFX complex and the RFX5(L66A) mutant: implications for the regulation of MHC class II gene expression. Biochemistry. 2007;46(6):1597–611. Epub 2007/02/07. 10.1021/bi6023868 . 17279624

[109] Lochamy J, Rogers EM, Boss JM. CREB and phospho-CREB interact with RFX5 and CIITA to regulate MHC class II genes. Mol Immunol. 2007;44(5):837–47. Epub 2006/05/30. 10.1016/j.molimm.2006.04.004 . 16730065

[110] Thakker S, Purushothaman P, Gupta N, Challa S, Cai Q, Verma SC. Kaposi's Sarcoma-Associated Herpesvirus Latency-Associated Nuclear Antigen Inhibits Major Histocompatibility Complex Class II Expression by Disrupting Enhanceosome Assembly through Binding with the Regulatory Factor X Complex. J Virol. 2015;89(10):5536–56. Epub 2015/03/06. 10.1128/JVI.03713-14 25740990 PMC4442538

[111] Boyd NH, Morgan JE, Greer SF. Polycomb recruitment at the Class II transactivator gene. Mol Immunol. 2015;67(2 Pt B):482–91. Epub 2015/08/19. 10.1016/j.molimm.2015.08.003 . 26283540

[112] Mehta NT, Truax AD, Boyd NH, Greer SF. Early epigenetic events regulate the adaptive immune response gene CIITA. Epigenetics. 2011;6(4):516–25. Epub 2011/01/27. 10.4161/epi.6.4.14516 . 21266852

[113] Guo M, Price MJ, Patterson DG, Barwick BG, Haines RR, Kania AK, et al EZH2 Represses the B Cell Transcriptional Program and Regulates Antibody-Secreting Cell Metabolism and Antibody Production. J Immunol. 2018;200(3):1039–52. Epub 2017/12/31. 10.4049/jimmunol.1701470 29288200 PMC5780247

[114] Hashwah H, Schmid CA, Kasser S, Bertram K, Stelling A, Manz MG, et al Inactivation of CREBBP expands the germinal center B cell compartment, down-regulates MHCII expression and promotes DLBCL growth. Proc Natl Acad Sci U S A. 2017;114(36):9701–6. Epub 2017/08/24. 10.1073/pnas.1619555114 28831000 PMC5594639

[115] Lin JH, Lin JY, Chou YC, Chen MR, Yeh TH, Lin CW, et al Epstein-Barr virus LMP2A suppresses MHC class II expression by regulating the B-cell transcription factors E47 and PU.1. Blood. 2015;125(14):2228–38. Epub 2015/01/30. 10.1182/blood-2014-08-594689 . 25631773

[116] Fujita N, Jaye DL, Geigerman C, Akyildiz A, Mooney MR, Boss JM, et al MTA3 and the Mi-2/NuRD complex regulate cell fate during B lymphocyte differentiation. Cell. 2004;119(1):75–86. Epub 2004/09/30. 10.1016/j.cell.2004.09.014 . 15454082

[117] Peng SL, Gerth AJ, Ranger AM, Glimcher LH. NFATc1 and NFATc2 together control both T and B cell activation and differentiation. Immunity. 2001;14(1):13–20. Epub 2001/02/13. 10.1016/s1074-7613(01)00085-1 . 11163226

[118] Tamura A, Hirai H, Yokota A, Kamio N, Sato A, Shoji T, et al C/EBPbeta is required for survival of Ly6C(-) monocytes. Blood. 2017;130(16):1809–18. Epub 2017/08/16. 10.1182/blood-2017-03-772962 28807982 PMC5649551

[119] Murn J, Mlinaric-Rascan I, Vaigot P, Alibert O, Frouin V, Gidrol X. A Myc-regulated transcriptional network controls B-cell fate in response to BCR triggering. BMC Genomics. 2009;10:323 Epub 2009/07/18. 10.1186/1471-2164-10-323 19607732 PMC2722676

[120] Habib T, Park H, Tsang M, de Alboran IM, Nicks A, Wilson L, et al Myc stimulates B lymphocyte differentiation and amplifies calcium signaling. J Cell Biol. 2007;179(4):717–31. Epub 2007/11/14. 10.1083/jcb.200704173 17998397 PMC2080907

[121] Rudolf R, Busch R, Patra AK, Muhammad K, Avots A, Andrau JC, et al Architecture and expression of the nfatc1 gene in lymphocytes. Front Immunol. 2014;5:21 Epub 2014/02/20. 10.3389/fimmu.2014.00021 24550910 PMC3909943

[122] Muhammad K, Rudolf R, Pham DAT, Klein-Hessling S, Takata K, Matsushita N, et al Induction of Short NFATc1/alphaA Isoform Interferes with Peripheral B Cell Differentiation. Front Immunol. 2018;9:32 Epub 2018/02/09. 10.3389/fimmu.2018.00032 29416540 PMC5787671

[123] Samitas K, Lotvall J, Bossios A. B cells: from early development to regulating allergic diseases. Arch Immunol Ther Exp (Warsz). 2010;58(3):209–25. Epub 2010/05/12. 10.1007/s00005-010-0073-2 . 20458549

[124] Miyazaki K, Miyazaki M, Murre C. The establishment of B versus T cell identity. Trends Immunol. 2014;35(5):205–10. Epub 2014/04/01. 10.1016/j.it.2014.02.009 24679436 PMC4030559

[125] Somasundaram R, Prasad MA, Ungerback J, Sigvardsson M. Transcription factor networks in B-cell differentiation link development to acute lymphoid leukemia. Blood. 2015;126(2):144–52. Epub 2015/05/21. 10.1182/blood-2014-12-575688 25990863 PMC4505011

[126] Hu Y, Yoshida T, Georgopoulos K. Transcriptional circuits in B cell transformation. Curr Opin Hematol. 2017;24(4):345–52. Epub 2017/05/04. 10.1097/MOH.0000000000000352 28463873 PMC5701664

[127] Schmitt C, Tonnelle C, Dalloul A, Chabannon C, Debre P, Rebollo A. Aiolos and Ikaros: regulators of lymphocyte development, homeostasis and lymphoproliferation. Apoptosis. 2002;7(3):277–84. Epub 2002/05/09. 10.1023/a:1015372322419 . 11997672

[128] Itoh-Nakadai A, Matsumoto M, Kato H, Sasaki J, Uehara Y, Sato Y, et al A Bach2-Cebp Gene Regulatory Network for the Commitment of Multipotent Hematopoietic Progenitors. Cell Rep. 2017;18(10):2401–14. Epub 2017/03/09. 10.1016/j.celrep.2017.02.029 . 28273455

[129] Ji Y, Lee HJ, Goodman C, Uskokovic M, Liby K, Sporn M, et al The synthetic triterpenoid CDDO-imidazolide induces monocytic differentiation by activating the Smad and ERK signaling pathways in HL60 leukemia cells. Mol Cancer Ther. 2006;5(6):1452–8. Epub 2006/07/05. 10.1158/1535-7163.MCT-06-0136 . 16818503

[130] Wang C, Petriello MC, Zhu B, Hennig B. PCB 126 induces monocyte/macrophage polarization and inflammation through AhR and NF-kappaB pathways. Toxicol Appl Pharmacol. 2019;367:71–81. Epub 2019/02/16. 10.1016/j.taap.2019.02.006 30768972 PMC6402591

[131] Hume DA. The Many Alternative Faces of Macrophage Activation. Front Immunol. 2015;6:370 Epub 2015/08/11. 10.3389/fimmu.2015.00370 26257737 PMC4510422

[132] Pierson E, Consortium GT, Koller D, Battle A, Mostafavi S, Ardlie KG, et al Sharing and Specificity of Co-expression Networks across 35 Human Tissues. PLoS Comput Biol. 2015;11(5):e1004220 Epub 2015/05/15. 10.1371/journal.pcbi.1004220 25970446 PMC4430528

[133] Medvedovic J, Ebert A, Tagoh H, Busslinger M. Pax5: a master regulator of B cell development and leukemogenesis. Adv Immunol. 2011;111:179–206. Epub 2011/10/06. 10.1016/B978-0-12-385991-4.00005-2 . 21970955

[134] Zhou L, Chong MM, Littman DR. Plasticity of CD4+ T cell lineage differentiation. Immunity. 2009;30(5):646–55. Epub 2009/05/26. 10.1016/j.immuni.2009.05.001 . 19464987

[135] Kyaw T, Toh BH, Bobik A. Foxp3+CD4+ Regulatory T-Cell Subtypes and Atherosclerosis. Circ Res. 2016;119(11):1151–3. Epub 2017/01/05. 10.1161/CIRCRESAHA.116.309999 . 28051776

[136] Kraj P, Ignatowicz L. The mechanisms shaping the repertoire of CD4(+) Foxp3(+) regulatory T cells. Immunology. 2018;153(3):290–6. Epub 2017/11/07. 10.1111/imm.12859 29106696 PMC5795179

[137] Pitard V, Roumanes D, Lafarge X, Couzi L, Garrigue I, Lafon ME, et al Long-term expansion of effector/memory Vdelta2-gammadelta T cells is a specific blood signature of CMV infection. Blood. 2008;112(4):1317–24. Epub 2008/06/10. 10.1182/blood-2008-01-136713 18539896 PMC2515135

[138] Farber DL, Yudanin NA, Restifo NP. Human memory T cells: generation, compartmentalization and homeostasis. Nat Rev Immunol. 2014;14(1):24–35. Epub 2013/12/18. 10.1038/nri3567 24336101 PMC4032067

[139] Angelo LS, Banerjee PP, Monaco-Shawver L, Rosen JB, Makedonas G, Forbes LR, et al Practical NK cell phenotyping and variability in healthy adults. Immunol Res. 2015;62(3):341–56. Epub 2015/05/28. 10.1007/s12026-015-8664-y 26013798 PMC4470870

[140] Boyette LB, Macedo C, Hadi K, Elinoff BD, Walters JT, Ramaswami B, et al Phenotype, function, and differentiation potential of human monocyte subsets. PLoS One. 2017;12(4):e0176460 Epub 2017/04/27. 10.1371/journal.pone.0176460 28445506 PMC5406034

[141] Rey-Giraud F, Hafner M, Ries CH. In vitro generation of monocyte-derived macrophages under serum-free conditions improves their tumor promoting functions. PLoS One. 2012;7(8):e42656 Epub 2012/08/11. 10.1371/journal.pone.0042656 22880072 PMC3412794

[142] Pang DJ, Neves JF, Sumaria N, Pennington DJ. Understanding the complexity of gammadelta T-cell subsets in mouse and human. Immunology. 2012;136(3):283–90. Epub 2012/03/06. 10.1111/j.1365-2567.2012.03582.x 22385416 PMC3385028

[143] Farag SS, Caligiuri MA. Human natural killer cell development and biology. Blood Rev. 2006;20(3):123–37. Epub 2005/12/21. 10.1016/j.blre.2005.10.001 . 16364519

