# Supplementary material for: A transcriptome-based approach to identify functional modules within and across primary human immune cells
Source: PLoS One. 2020 May 29;15(5):e0233543. doi: 10.1371/journal.pone.0233543 (PMC7259617; doi:10.1371/journal.pone.0233543)
Supplement: S1 Text — (DOCX) [file pone.0233543.s001.docx]

**S1 Text. Analysis of all no-TEG and all no-MRG genes in *modules 22, 38 and 41***

To validate our approach of global and targeted functional annotation of co-expression modules, the entire list of genes within ***modules 22, 38 and 41***, were analyzed using the GeneCards, PubMed and Google Scholar databases (see Materials and Methods section).

- **B lymphocyte-specific modules *22 and 38*; cell activation and BCR receptor engagements.** As described in the main text, global analysis of ***module 22*** showed gene annotation enrichment for specific B cell functions and target analyses revealed genes involved in B cell receptor (BCR) structure and/or signaling and B cell activation, development and maturation. For ***module 38***, no specific functions were identified via gene annotation enrichment analysis, but the targeted analyses identified genes involved in BCR activation, Ig assembly, and B cell signaling. Extending the annotation analyses to the entire set of genes in ***module 22*** identified encoding genes participating in the functions identified by the global and targeted analyses presented above. For example, the *BLK* gene, which is part of the complete list of genes in ***module 22*** but was not highlighted in the targeted analyses, is known to interact with BANK1 and CD79A in order to link BCR-mediated signaling to the formation of intracellular second messengers through PLCG2 [1]. In addition, many other genes were involved in BCR stimulation (*CD19, FCRL1, FCRL2, MS5A1,* and *TEAD2*). These functions are presented in **Figure 4**. Some genes in ***module 22*** were implicated in apoptosis (*CD24, CDCA7L, DNASE1L3, RASSF6, SYVN1,* and *TP63*), Ig presentation and expression (*FCER2, IL4R,* and *MZB1*), B cell differentiation (*CXCR5* and *FCER2*) and Wnt signaling pathway (*CSNK1G3* and *ROR1*). These analyses suggest that one of the primary functions of the genes in ***module 22*** is B cell activation via the BCR signaling pathway. Extended analysis of ***module 38*** genes identified the classical B-cell genes *EBF1*, a transcriptional activator, and *IL6*, an inflammatory cytokine. Moreover, this module contains numerous genes involved in endosome/vesicle trafficking (*ACBD3, AP1S3, MARCH3,* and *USP6NL*), actin/cytoskeleton remodeling (*ARHGAP20, DMD,* and *ENAH*), and lipid metabolism (*ABCB4, FA2H,* and *SMPD2*). Although these have not been studied in B cells, they could potentially play a role in events pre- and post- engagement of the BCR such as endocytosis, signaling, and formation of lipid rafts. Taken together, these analyses suggest that ***module 38*** genes associated with the events that surround BCR receptor engagement and related pathways.
- ***Module 41* shared by B lymphocytes and Monocytes; a gene expression program enabling antigen processing and presentation.** Global analysis of the 39 genes within this module revealed enrichments significantly enriched, with the three most significant (%FDR <10^-8^) being “endosome”, “MHC classes I/II-like antigen recognition protein”, and “MHC class II protein complex”. Targeted analyses found a core set of genes that clearly highlights a central role in antigen processing and presentation. When exploring the remaining genes within this module, additional HLA class II paralogues can be found (*HLA-DQA1* and *HLA-DRB5*), as well as a gene that encodes the protein SCIMP, which is involved in HLA class II signal transduction and immune synapse formation [2, 3]. In addition, this module contains a number of genes that encode proteins involved in vesicular traffic (*BLOC1S7, LAMP5, TBC1D5,* and *TRAK1*), potentially revealing novel players in the trafficking of endosomal vesicles involved in antigen processing and presentation. Although to a much lesser extent, there is also evidence that this module may also be involved in non-specific immune recognition (*CD180* and *CLEC4G*) and signaling (*LY86, GCNT1* and *IRAK1*) in response to exposure to pathogens [4, 5].
