# Supplementary material for: A transcriptome-based approach to identify functional modules within and across primary human immune cells
Source: PLoS One. 2020 May 29;15(5):e0233543. doi: 10.1371/journal.pone.0233543 (PMC7259617; doi:10.1371/journal.pone.0233543)
Supplement: S2 Text — (DOCX) [file pone.0233543.s002.docx]

**S2 Text. Additional analyses of genes highly-expressed in Neutrophils**.

The distinctive nature of the neutrophils was also observed in the co-expression analyses, with 13 of the 45 co-expression ***modules (4, 7, 10-12, 14-16, 21, 25, 35, 36, 40)*** highly associated with neutrophils (**Fig 3**); six of which were essentially exclusively associated with the neutrophils (***modules 11, 14-16, 21, 40***), whereas the others were also associated with other myeloid populations (monocytes and/or macrophages and/or LPS-activated macrophages). This is also reflected by a number of specific transcripts that are very different between these groups. In fact, there were 15 transcripts with a greater than 100-fold difference between the average number of reads in neutrophils compared to the average number of reads across all other cell types combined: *TRPM6, CA4, LOC101929866, NFE4, BMX, ALPL, LOC102724662, FCGR3B, CXCR1, CXCR2, TNFRSF10C, NECAB2, MME, MMP25,* and *CSNK1A1L*. Nine of these (*underlined*) are part of ***module 11***, the module most associated with neutrophils. Many of these transcripts encode proteins that are hallmarks of neutrophils: *ALPL* (encoding the tissue non-specific alkaline phosphatase believed to play a critical role in the anti-microbial function of neutrophils by promoting its migration and ROS generation), *FCGR3B*, (low-affinity receptor for the Fc region of gamma immunoglobulins (IgG)) *CXCR1* and *CXCR2* (encoding for the receptor the interleukin 8), *TNFRSF10C* (encoding the receptor for TRAIL and involved in neutrophil apoptosis), *MME* (encoding CD10 which is present on the membrane of neutrophilic secretory granules), and *MMP25* (encoding Matrix Metallopeptidase 25, which is key to trans-endothelial migration of neutrophils to inflammatory sites) [6-10].

The neutrophil-related functions encoded by the other genes in this list are not as well characterized. Specifically, *TRPM6* encodes the enzyme-coupled transient receptor potential channel subfamily M member 6 that, along with its homolog *TRPM7*, are important for Mg2+ homeostasis but are distinct from all other ion channels in that they also have kinase domains [11]. While Mg2+ is certainly critical to the function of multiple enzymes, these channels’ ability to conduct other divalent cations such as calcium, as well as their kinase activities, supports an even broader impact [11]. For example, it has been shown that TRPM7 regulates intracellular calcium signals in T cells as well as macrophage activation and polarization [12]. While the role of *TRPM6* in the immune system is unknown, it’s a high expression in neutrophils and absence in the other immune cells tested here suggested a neutrophil-specific role, which may include neutrophil chemotaxis given its interaction with *RACK1* (alias *GNB2L1*). CA4 encodes the carbonic anhydrase 4 and is responsible for catalyzing the reversible hydration of carbon dioxide to form bicarbonate (HCO3-) and hydrogen (H+) ions, and is suspected in having a role in the regulation of neutrophil activity and wound healing [13, 14]. *NFE4* nuclear factor is involved in human globin gene switching in fetal erythroid cells [15], but may also have a role in transcriptional regulation of neutrophil genes. *BMX* encodes a non-receptor tyrosine kinase that plays central but diverse modulatory roles in various signaling processes in different cell types, with a likely role the regulation of the functional responsiveness of neutrophils to chemotactic factors [16]. *NECAB2*, primarily studied as a neuronal calcium binding protein may regulate important neutrophil functions given its ability to modulate the adenosine A(2A) receptor (alias *ADORA2A*) and the latter’s role in neutrophil apoptosis, autophagy and NETs [17-19]. *CSNK1A1L* (Casein Kinase 1 Alpha 1 Like) is a poorly characterized member of the CK1 protein family involved in multiple functions likely mediated by its broad serine/threonine-protein kinase activity, including host defense response against infectious pathogens. For example, when CK1alpha phosphorylates type I interferon receptor 1 (*IFNAR1*) at Ser535 thereby induces the latter’s ubiquitination and degradation [20].
