# Supplementary material for: A transcriptome-based approach to identify functional modules within and across primary human immune cells
Source: PLoS One. 2020 May 29;15(5):e0233543. doi: 10.1371/journal.pone.0233543 (PMC7259617; doi:10.1371/journal.pone.0233543)
Supplement: S1 Fig — Results are expressed as median percentage among CD3+CD4+ (n = 12), CD3+CD8+ (n = 12) or CD3+ TCRγδ+ (n = 8) T lymphocyte populations respectively and among CD3-CD19+ B lymphocyte (n = 12), CD3-CD56+ NK cell (n = 12) or CD14+ monocyte (n = 12) populations, with quartiles [Q1-Q3] for each subset. CD14+ enriched mononuclear cells were stimulated in vitro with M-CSF in order to obtain macrophages, with an additional 24h LPS stimulation to obtain activated macrophages. Cells were stained with the mentioned surface markers and analyzed by flow cytometry. Results presented are the median MFI of specific staining minus unstained with quartiles [Q1-Q3]. [9, 95, 137–143]. (DOCX) [file pone.0233543.s003.docx]

**
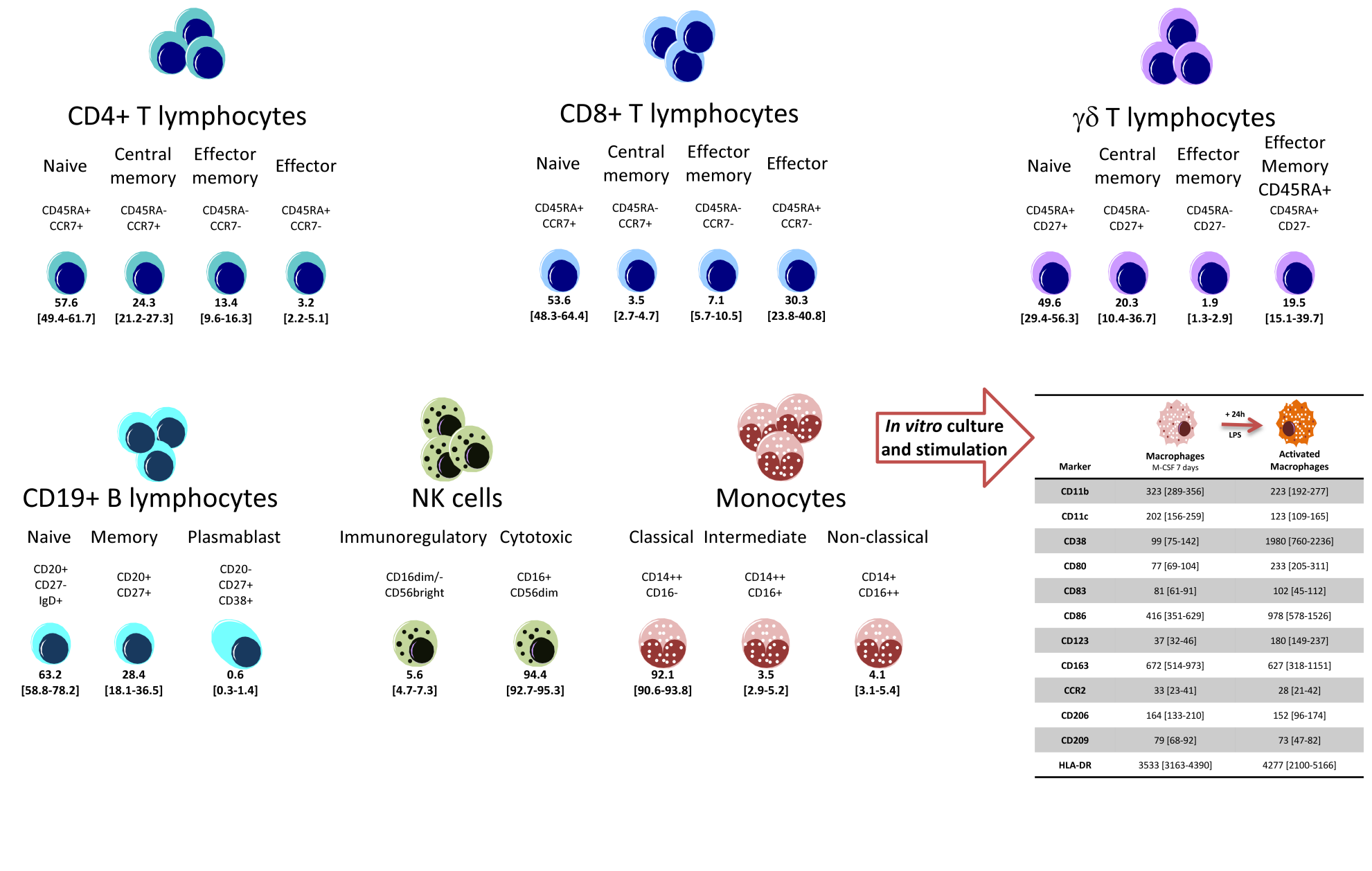
**

**S1 Fig. Immunophenotyping of immune cell populations from human peripheral blood**. Results are expressed as median percentage among CD3+CD4+ (n=12), CD3+CD8+ (n=12) or CD3+ TCRγδ+ (n=8) T lymphocyte populations respectively and among CD3-CD19+ B lymphocyte (n=12), CD3-CD56+ NK cell (n=12) or CD14+ monocyte (n=12) populations, with quartiles [Q1-Q3] for each subset. CD14+ enriched mononuclear cells were stimulated *in vitro with* M-CSF in order to obtain macrophages, with an additional 24h LPS stimulation to obtain activated macrophages. Cells were stained with the mentioned surface markers and analyzed by flow cytometry. Results presented are the median MFI of specific staining minus unstained with quartiles [Q1-Q3]. [21-29].
