# Supplementary material for: A transcriptome-based approach to identify functional modules within and across primary human immune cells
Source: PLoS One. 2020 May 29;15(5):e0233543. doi: 10.1371/journal.pone.0233543 (PMC7259617; doi:10.1371/journal.pone.0233543)
Supplement: S2 Fig — Each boxplot represents the mean of gene expression in log2 for a cell type in our 12 individuals. Panel A shows the log2 of cpm normalized by library size and TMM normalization factor. Panel B shows the distribution after variance stabilization by R function voom. Even after normalization with voom, a function of Limma package, the median of neutrophil gene distribution is not aligned with other cell types because many more genes are not expressed in neutrophils than other primary immune cell types in our data [29]. (DOCX) [file pone.0233543.s004.docx]

**
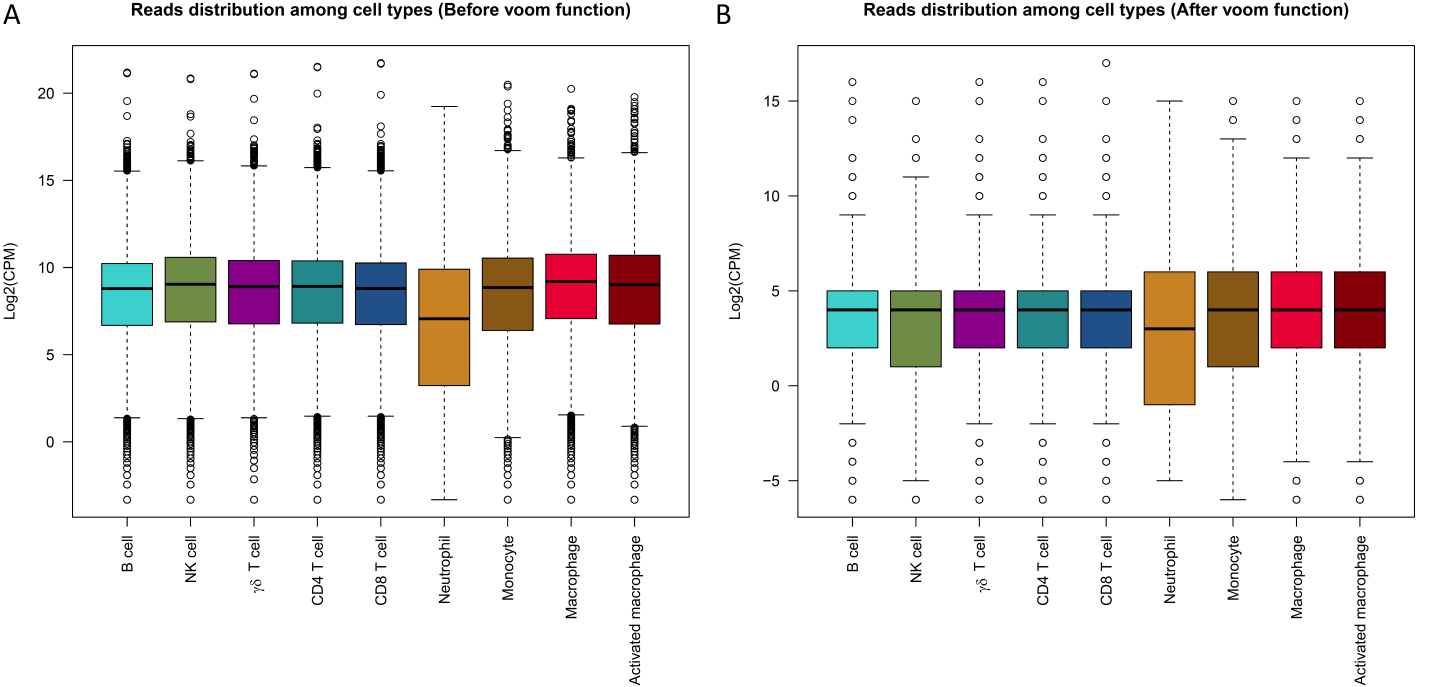
 S2 Fig.** **Boxplot of gene expression distribution after normalization before and after normalization.** Each boxplot represents the mean of gene expression in log2 for a cell type in our 12 individuals. Panel A shows the log2 of cpm normalized by library size and TMM normalization factor. Panel B shows the distribution after variance stabilization by R function *voom*. Even after normalization with *voom*, a function of Limma package, the median of neutrophil gene distribution is not aligned with other cell types because many more genes are not expressed in neutrophils than other primary immune cell types in our data [30].
