# Supplementary material for: A transcriptome-based approach to identify functional modules within and across primary human immune cells
Source: PLoS One. 2020 May 29;15(5):e0233543. doi: 10.1371/journal.pone.0233543 (PMC7259617; doi:10.1371/journal.pone.0233543)
Supplement: S3 Fig — Scale independence and mean value connectivity were used to select the β parameter, an exponent to the gene correlation matrix that determines the emphasis put on higher vs lower correlations [34]. Value 12 was chosen for β because of it as a good trade-off between scale-free topology (R^2 = 0.719) and connectivity. Therefore, β = 12 was used to compute dissimilarities between genes with the WGCNA functions, adjacency and TomsimilarityFromExpr (Topology Overlap Matrix Similarity From Expression) [33, 34]. The red line on scale independence graph represents value 0.8 (suggested by the authors). (DOCX) [file pone.0233543.s005.docx]

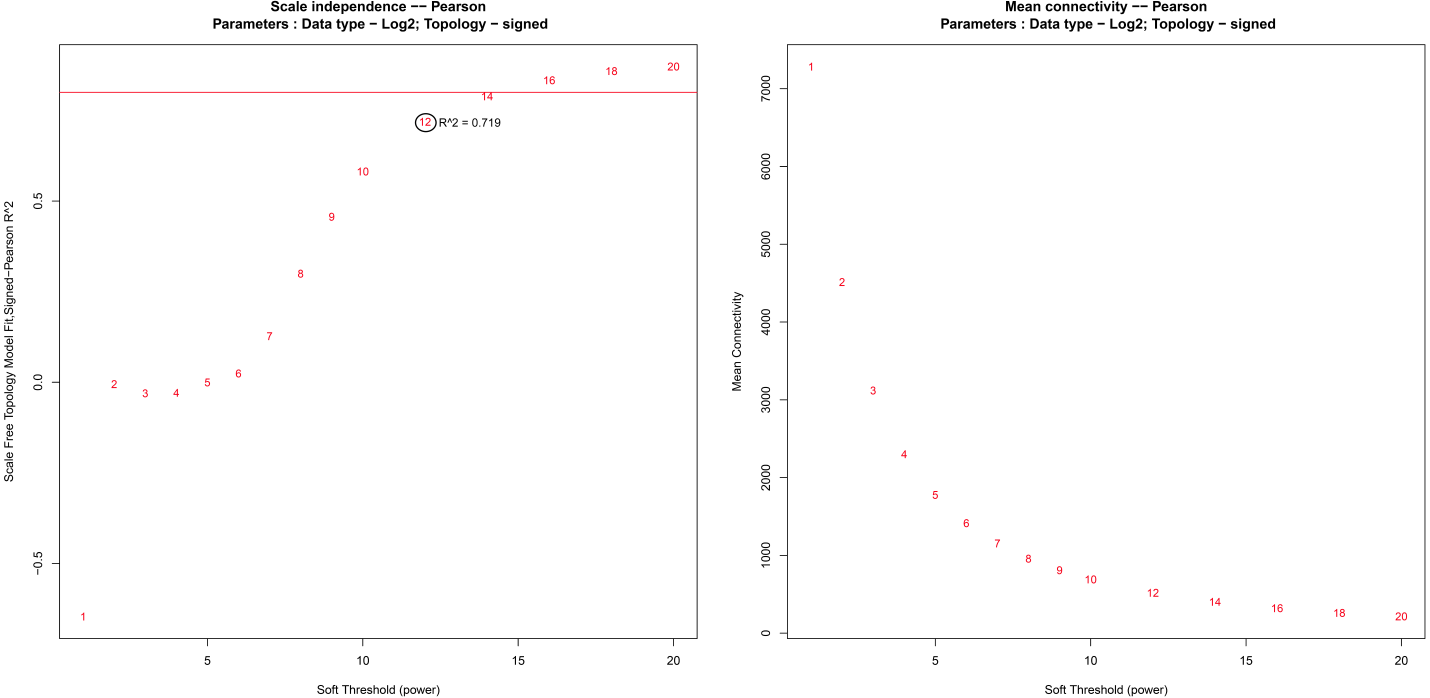


**S3 Fig.** **Plot of scale independence and mean connectivity – Pearson correlation.** Scale independence and mean value connectivity were used to select the β parameter, an exponent to the gene correlation matrix that determines the emphasis put on higher vs lower correlations [31]. Value 12 was chosen for β because of it as a good trade-off between scale-free topology (R^2 = 0.719) and connectivity. Therefore, β=12 was used to compute dissimilarities between genes with the WGCNA functions, *adjacency* and *TomsimilarityFromExpr* (Topology Overlap Matrix Similarity From Expression) [31, 32]. The red line on scale independence graph represents value 0.8 (suggested by the authors).
