# Supplementary material for: A transcriptome-based approach to identify functional modules within and across primary human immune cells
Source: PLoS One. 2020 May 29;15(5):e0233543. doi: 10.1371/journal.pone.0233543 (PMC7259617; doi:10.1371/journal.pone.0233543)
Supplement: S4 Fig — Graph A represents the tree of modules obtained with WGCNA tools. The red line on this graph is the value 0.05 who chooses to cut the tree to grouping similar modules in one. Graph B represents the new modules after cuts tree with new numeration. (DOCX) [file pone.0233543.s006.docx]

**
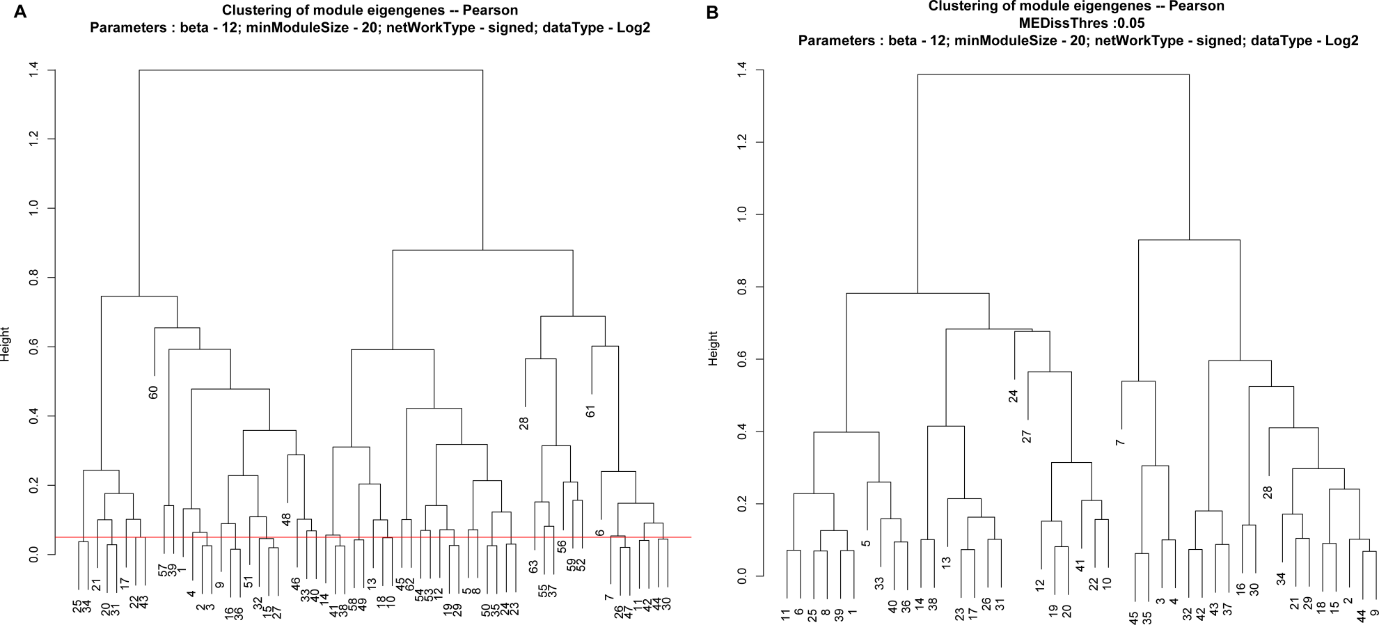
**

**S4 Fig.** **Hierarchical trees of gene modules before and after cut tree.** Graph A represents the tree of modules obtained with WGCNA tool. The red line on this graph is the value 0.05 who chooses to cut the tree to grouping similar modules in one. Graph B represents the new modules after cuts tree with new numeration.
