# Supplementary material for: A transcriptome-based approach to identify functional modules within and across primary human immune cells
Source: PLoS One. 2020 May 29;15(5):e0233543. doi: 10.1371/journal.pone.0233543 (PMC7259617; doi:10.1371/journal.pone.0233543)
Supplement: S5 Fig — To identify transcription factors consistent with having a role in cell fate decisions we examined differential gene expression for all known human transcription factors (n = 1638) [44]. Schematic simplification is used as a representation of hematopoiesis from lymphoid and myeloid lineage. Transcription factors are in red and black. Red represents transcription factors known to be involved in the establishment and/or maintaining cell/lineage differentiation. The pink background color is used for transcription factors associated with cytotoxic cells. Blue arrows show increased or decreased expression of genes coding for transcription factors. Complete list of candidate TFs in S5 Table (DOCX) [file pone.0233543.s007.docx]

**
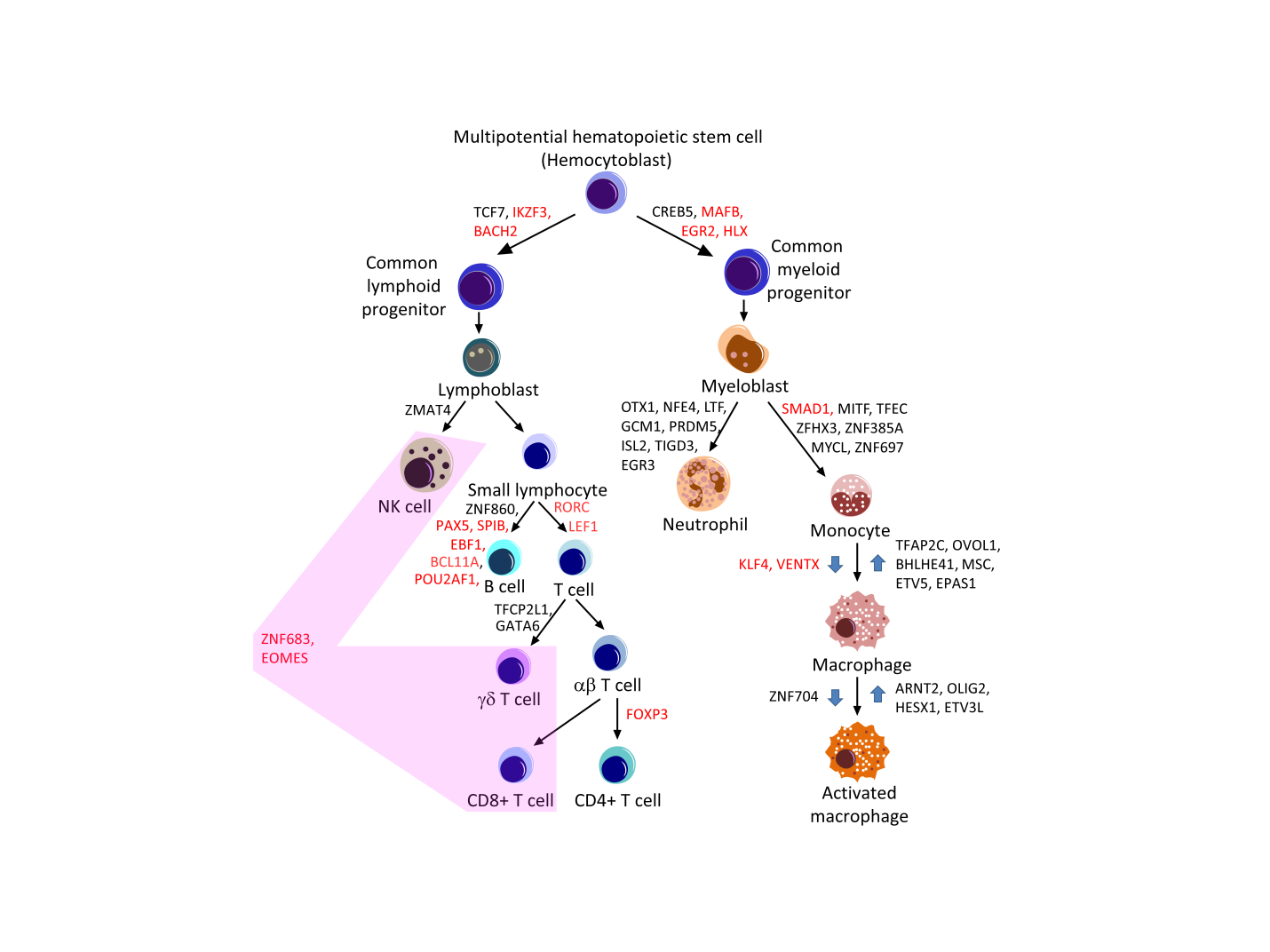
**

**S5 Fig.** **Hematopoietic differentiation scheme and associated transcription factors from differential gene expression.** To identify transcription factors consistent with having a role in cell fate decisions we examined differential gene expression for all known human transcription factors (n=1638) [33]. Schematic simplification is used as a representation of hematopoiesis from lymphoid and myeloid lineage. Transcription factors are in red and black. Red represents transcription factors known to be involved in the establishment and/or maintaining cell/lineage differentiation. The pink background color is used for transcription factors associated with cytotoxic cells. Blue arrows show increased or decreased expression of genes coding for transcription factors. Complete list of candidate TFs in **Table S5**.
