# Supplementary material for: A transcriptome-based approach to identify functional modules within and across primary human immune cells
Source: PLoS One. 2020 May 29;15(5):e0233543. doi: 10.1371/journal.pone.0233543 (PMC7259617; doi:10.1371/journal.pone.0233543)
Supplement: S7 Fig — The heatmap represents gene normalized expression levels (log2 of cpm) in our nine cell types. Red is the higher value and yellow, the lower. (DOCX) [file pone.0233543.s009.docx]

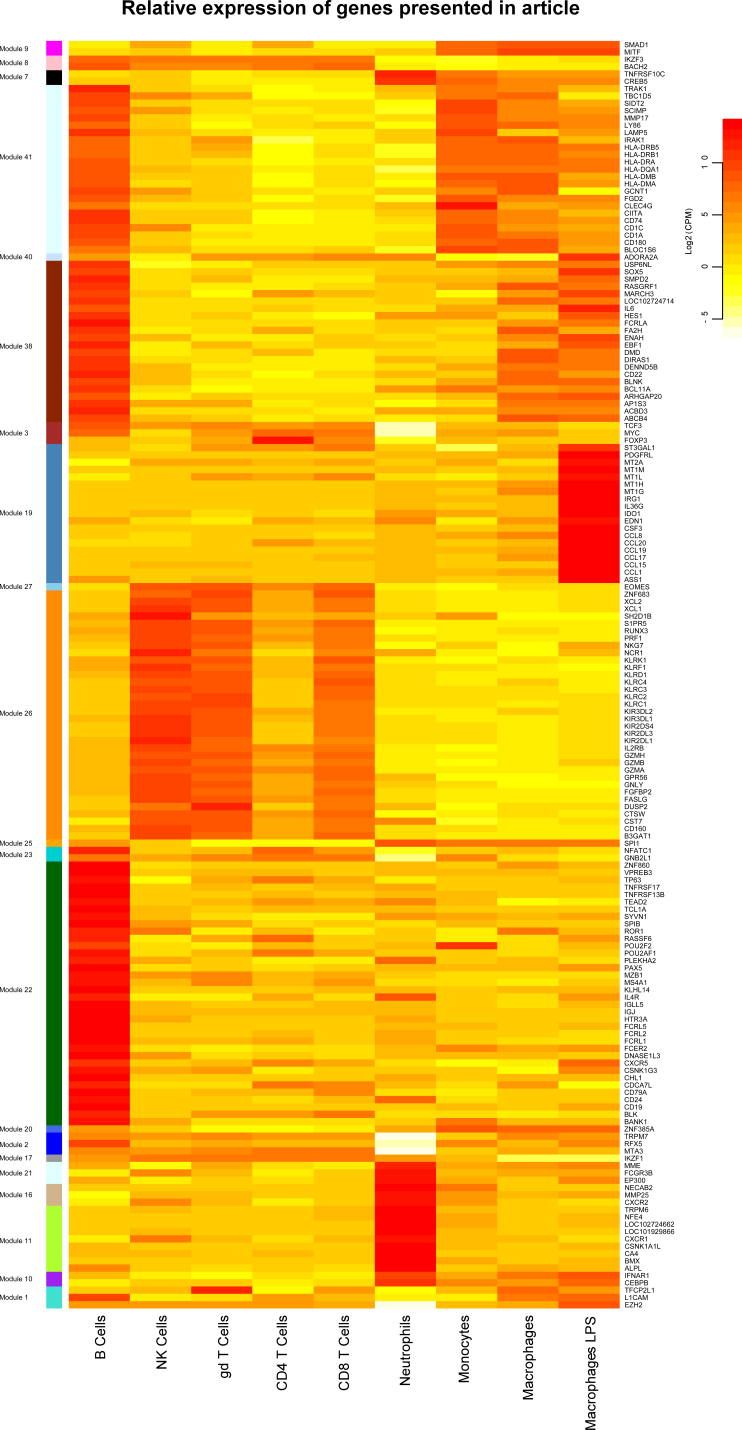
**S7 Fig. Heatmap of mean normalized expression for a subset of genes.** The heatmap represents gene normalized expression levels in our nine cell types. Red is the higher value and yellow, the lower.
