# Supplementary material for: A transcriptome-based approach to identify functional modules within and across primary human immune cells
Source: PLoS One. 2020 May 29;15(5):e0233543. doi: 10.1371/journal.pone.0233543 (PMC7259617; doi:10.1371/journal.pone.0233543)
Supplement: S8 Fig — Global and targeted analyses of the genes within module 41 were primarily associated with the presentation of peptide and lipid antigens. Genes in module 41 are represented in orange: Top Expressing Genes in dark orange, Module Representative Genes in intermediate orange and other genes in light orange. Genes from this module act together to establish Major Histocompatibility Complex class II function. To see the profile of gene expression mean of all genes of module 41 presented in this figure refers to the heatmap in S7 Fig. (DOCX) [file pone.0233543.s010.docx]

**
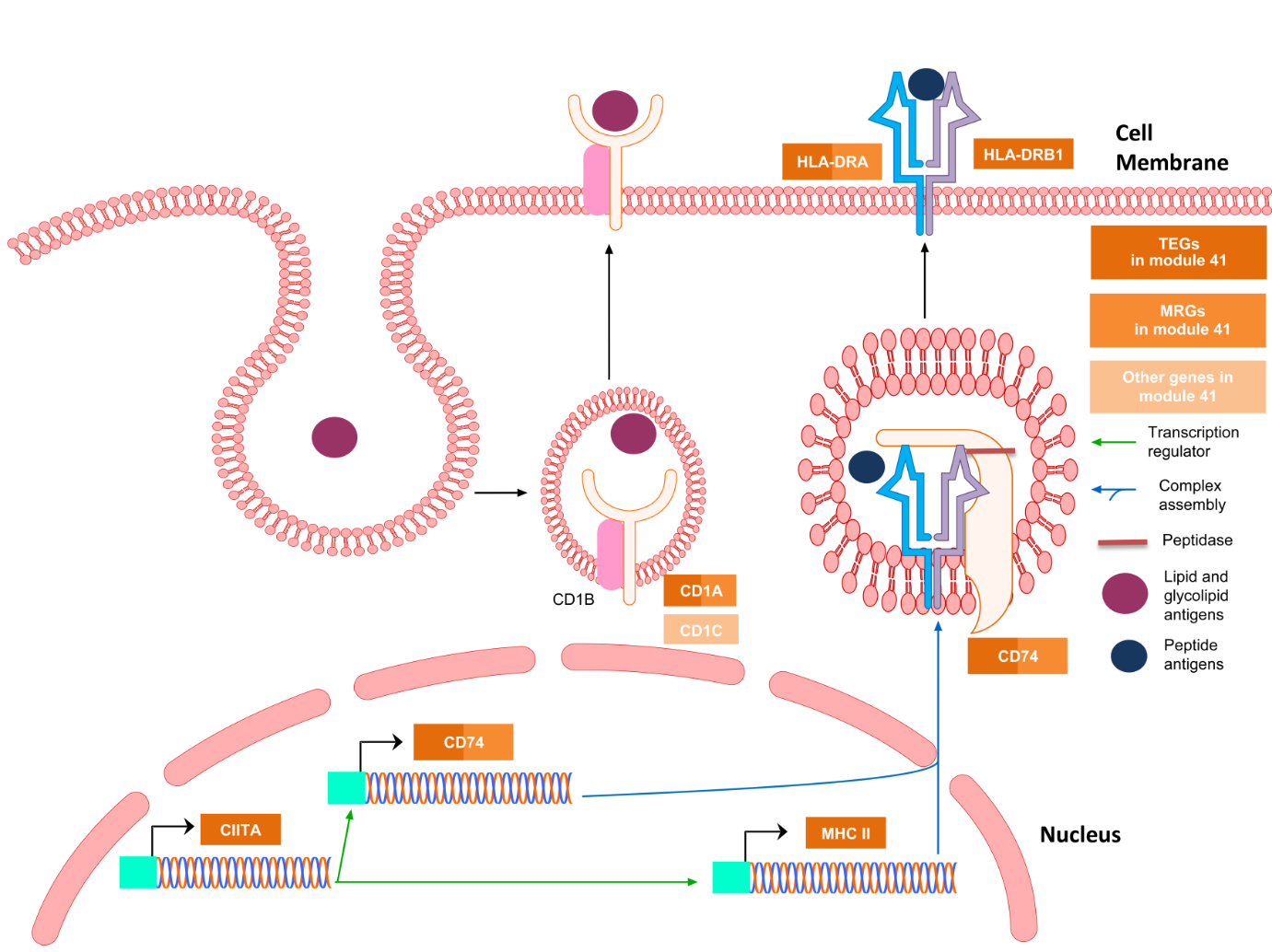
**

**S8 Fig. Global and targeted** a**nalyses of** **genes within module 41, associated with B cells and monocytes, describe MHC class II and antigen processing and presentation functions.** Global and targeted analyses of the genes within *module 41* were primarily associated with the presentation of peptide and lipid antigens. Genes in module 41 are represented in orange: *Top Expressing Genes* in dark orange, *Module Representative Genes* in intermediate orange and other genes in light orange. Genes from this module act together to establish Major Histocompatibility Complex class II function. To see the profile of gene expression mean of all genes of module 41 presented in this figure refers to the heatmap in **S7 Fig**.
