# Supplementary material for: A transcriptome-based approach to identify functional modules within and across primary human immune cells
Source: PLoS One. 2020 May 29;15(5):e0233543. doi: 10.1371/journal.pone.0233543 (PMC7259617; doi:10.1371/journal.pone.0233543)
Supplement: S1 Table — (DOCX) [file pone.0233543.s011.docx]

**S1 Table.** **List of antibodies used for lymphocyte/monocyte immunophenotyping.**

| **Antigen** | **Antibody clone** | **Supplier** | **Antigen** | **Antibody clone** | **Supplier** |
| --- | --- | --- | --- | --- | --- |
| CD3 | UCHT1 | Biolegend | CD27 | O323 | Biolegend |
| CD4 | RPA-T4 | Biolegend | CD38 | HIT2 | Biolegend |
| CD8 | RPA-T8 | Biolegend | CD45RA | HI100 | Biolegend |
| CD14 | M5E2 | Biolegend | CD56 | B159 | BD Biosciences |
| CD16 | 3G8 | Biolegend | CCR7 (CD197) | 150503 | BD Biosciences |
| CD19 | HIB19 | Biolegend | IgD | IA6-2 | Biolegend |
| CD20 | 2H7 | Biolegend | TCRγδ | B1 | Biolegend |
