# Supplementary material for: A transcriptome-based approach to identify functional modules within and across primary human immune cells
Source: PLoS One. 2020 May 29;15(5):e0233543. doi: 10.1371/journal.pone.0233543 (PMC7259617; doi:10.1371/journal.pone.0233543)
Supplement: S2 Table — (DOCX) [file pone.0233543.s012.docx]

**S2 Table.** **List of antibodies used for macrophage immunophenotyping.**

| **Antigen** | **Antibody clone** | **Supplier** | **Antigen** | **Antibody clone** | **Supplier** |
| --- | --- | --- | --- | --- | --- |
| CD11b | ICRF44 | Biolegend | CD123 | 6H6 | Biolegend |
| CD11c | 3.9 | Biolegend | CD163 | GHI/61 | Biolegend |
| CD38 | HIT2 | Biolegend | CCR2 (CD192) | TG5/CCR2 | Biolegend |
| CD80 | 2D10 | Biolegend | CD206 | 15-2 | Biolegend |
| CD83 | HB15e | Biolegend | CD209 | 9E9A8 | Biolegend |
| CD86 | IT2.2 | Biolegend | HLA-DR | L243 | Biolegend |
