# Supplementary material for: A transcriptome-based approach to identify functional modules within and across primary human immune cells
Source: PLoS One. 2020 May 29;15(5):e0233543. doi: 10.1371/journal.pone.0233543 (PMC7259617; doi:10.1371/journal.pone.0233543)
Supplement: S3 Table — Values are reads at each step. (DOCX) [file pone.0233543.s013.docx]

**S3 Table.** **Summary statistics of RNA-Seq data from raw reads through quality control steps.** Values are reads at each step.

|  | **Monocytes** | | **Macrophages** | | **Activated**  **macrophages** | | **Neutrophils** | |
| --- | --- | --- | --- | --- | --- | --- | --- | --- |
| # of samples | 12 | | 12 | | 12 | | 11 | |
|  | | | | | | | | |
|  | **Mean** | **Std. Dev.** | **Mean** | **Std. Dev.** | **Mean** | **Std. Dev.** | **Mean** | **Std. Dev.** |
| Raw FASTQ | 25392853 | 2971647 | 25848399 | 4000125 | 26306327 | 4031458 | 26977675 | 4477712 |
| Filtered FASTQ | 24841788 | 2849773 | 25331060 | 3910406 | 25769770 | 3942984 | 26380553 | 4329742 |
| Raw alignments | 23926839 | 2633409 | 24215782 | 3732856 | 24610852 | 3742637 | 25275616 | 4183719 |
| Properly paired | 21001032 | 2349963 | 21533824 | 3318728 | 21872887 | 3497270 | 22082978 | 3558045 |

|  | **B cell** | | **CD4+ T cell** | | **CD8+ T cell** | | **γδ T cell** | | **NK cell** | |
| --- | --- | --- | --- | --- | --- | --- | --- | --- | --- | --- |
| # of samples | 12 | | 12 | | 12 | | 8 | | 12 | |
|  | | | | | | | | | | |
|  | **Mean** | **Std. Dev.** | **Mean** | **Std. Dev.** | **Mean** | **Std. Dev.** | **Mean** | **Std. Dev.** | **Mean** | **Std. Dev.** |
| Raw FASTQ | 23163724 | 2500054 | 24239648 | 3610972 | 23447063 | 2740439 | 23816789 | 2479157 | 24248847 | 2453890 |
| Filtered FASTQ | 22670510 | 2402592 | 23737575 | 3517709 | 22941165 | 2612434 | 23325587 | 2399476 | 23727620 | 2359340 |
| Raw alignments | 22010211 | 2252667 | 23180233 | 3572610 | 22493549 | 2803995 | 22597219 | 2371193 | 22942354 | 2253393 |
| Properly paired | 19050284 | 1894288 | 20358730 | 2976246 | 19611247 | 2172733 | 19908136 | 1998188 | 20207296 | 1904528 |
