# Supplementary material for: A transcriptome-based approach to identify functional modules within and across primary human immune cells
Source: PLoS One. 2020 May 29;15(5):e0233543. doi: 10.1371/journal.pone.0233543 (PMC7259617; doi:10.1371/journal.pone.0233543)
Supplement: S8 Table — *** The TFs IRF4, PAX5, and BACH2, along with the absence of BCL6, have been reported to also play a role in the maturation of mature naïve to memory B cells. (26751566); Number in parenthesis is PMID number, which is the unique identifier number used in PubMed for each article. (DOCX) [file pone.0233543.s018.docx]

**S8 Table.** **Literature review of key transcription factors involved in B-cell differentiation and maturation.**

| **Cell types/**  **lineages** | **Known in B cell differentiation** | **Known in mature B cell** | **Known in Both** | **TF Gene Symbol** | **Literature** |
| --- | --- | --- | --- | --- | --- |
| B-cell-lineage differentiation CLP to mature naïve B cell | √ | √ | √ | BACH2*** | BACH2 inhibits myeloid differentiation in common lymphoid progenitors. (28273455, 25990863) BACH2 is involved in V(D)J recombination in pre-B cell and its expression locks differentiation of human mature B cells to plasma cells. (23852341, 28359033, 29129929, 24602812, 26751566) |
|  | √ | √ | √ | EBF1 | EBF1 and FOXO1 expression establish B cell identity and they activate the expression of PAX5 in early pro-B cells, coordinately with TCF3. (24679436) EBF1 is a key transcription factor of B cell specification and commitment, EBF1 is implicated in establishing and maintaining B-cell identity, EBF1 is also required to coordinate differentiation with cell proliferation and survival (25123279) |
|  | √ | √ | √ | PAX5*** | PAX5 is essential for the commitment of lymphoid progenitors to the B lymphocyte lineage. PAX5 represses B lineage inappropriate genes and activates B lineage-specific genes. PAX5 involved in the identity and function of B cell throughout B lymphopoiesis and in naive and memory B cells. (21970955, 17440452, 26751566) |
|  | √ | √ | √ | POU2AF1 | With POU2F2 or POU2F1, POU2AF1 plays a role in B cell proliferation, induces surface marker expression during B-cell differentiation and enables B cells to respond normally to antigen receptor signals (24061476, 24688485, 19104664) |
|  | √ | √ | √ | SPIB | SPIB enables B cells to appropriately respond to environmental cues and is a direct target of POU2AF1 (29127283, 16861304). SPI1 and SPIB are essential transcriptional regulators of B-cell differentiation (21768304) |
|  | √ | √ | √ | BCLL11A | BCL11A is essential for lymphoid development and negatively regulates p53 that inhibited apoptosis. (23230003; 24648892) |
|  | √ | √ | √ | TCF3 | TCF3 plays a particularly important role in B-lineage specification and involved in FOXO1, EBF1, and PAX5 regulation. (24679436, 27261530, 18538592) TCF3 is activated during BCR signaling^.^ (26081581) IKZF1, SPI1, and TCF3 play a role in functional lymphoid lineage priming. (25990863) |
|  | √ | √ | √ | IKZF1 | IKZF1, SPI1, and TCF3 play a role in functional lymphoid lineage priming. IKZF1 has a role in the formation of pre-BCR in the pre-B cell stage (25990863, 23303821) IKZF1 limit the response of naïve splenic B cells to BCR signals and is a negative regulator of follicular B cell activation. (26775846) |
| Necessary but not sufficient in B-cell-lineage differentiation | √ | √ | √ | CTCF | CTCF modifies DNA structures by enhancer-promoter interactions that involve lineage-specific transcriptional regulators such as TCF3, EBF1, and FOXO1. (24679436) |
|  | √ | √ | √ | SPI1 | IKZF1, SPI1, and TCF3 play a role in functional lymphoid lineage priming. (25990863) With IRF4 or IRF8, SPI1 involves pre-B and early-B cell development and they suppress pre-B-cell leukemia associated with reduced expression of the established B-lineage tumor suppressor genes, IKZF1 and SPIB. (26932576) |
|  | √ | √ | √ | RUNX1 | RUNX1can regulate B-cell growth. (16584381, 25205721) |
|  | √ | √ | √ | POU2F2 | With POU2AF1, POU2F2 plays a role in B cell proliferation, induces surface marker expression during B-cell differentiation and enables B cells to respond normally to antigen receptor signals (24061476, 24688485, 19104664) |
|  | √ | √ | √ | FOXO1 | With TCF3, FOXO1 induces the expression of EBF1 in pre-pro-B cell and they activate the expression of PAX5 in early pro-B cells. FOXO1 and EBF1 establish B cell identity. (24679436) FOXO1 promotes differentiation, proliferation, survival, immunoglobulin gene rearrangement, and class switching in B cells. (29431075) |
|  | √ | √ | √ | IRF4*** | With SPI1, IRF4 involved in pre-B and early-B cell development and they suppress pre-B-cell leukemia associated with reduced expression of the established B-lineage tumor suppressor genes, IKZF1 and SPIB. (26932576) PRDM1 and IRF4 are essential for the generation of plasma cells. IRF4 is essential for the survival of plasma cells. (26779600) |
|  | √ | √ | √ | IRF8 | With SPI1, IRF8 involved in early-B-cell development and of recirculating B-cell numbers. They restore IKZF1 and SPIB gene expression in pre-B cells to inhibit leukemic cell growth. (26932576) IRF8 and SPI1 complex negatively regulates plasma cell differentiation by concurrently promoting the expression of BCL6 and PAX5 and repressing AID and PRDM1. (25288399) |
| Naïve B cell to plasma cell |  | √ |  | PRDM1 | PRDM1 acts as a master regulator of plasma cell differentiation. (25115512) PRDM1 and IRF4 are essential for the generation of plasma cells. PRDM1 regulates the transcriptional identity of plasma cells, whose XBP1 expression. PRDM1 is also involved in antibody secretion by plasma cells. (26779600, 15345222) |
|  |  | √ |  | XBP1 | XBP1 expression is regulated by PRDM1 in plasma cells. (26779600) XBP1 is selectively and specifically required to initiate and maintaining plasma-cell differentiation from mature B cells. (11460154) XBP1 is involved in secretory plasma cell functions such as cell size, lysosome content, mitochondrial mass and function, ribosome numbers, and total protein synthesis. (15345222) |

*** The TFs IRF4, PAX5, and BACH2, along with the absence of BCL6, have been reported to also play a role in the maturation of mature naïve to memory B cells. (26751566); Number in parenthesis is PMID number, which is the unique identifier number used in PubMed for each article.
