# Supplementary material for: A transcriptome-based approach to identify functional modules within and across primary human immune cells
Source: PLoS One. 2020 May 29;15(5):e0233543. doi: 10.1371/journal.pone.0233543 (PMC7259617; doi:10.1371/journal.pone.0233543)
Supplement: S1 Data — (DOC) [file pone.0233543.s019.doc]

**Supplementary references**

1. Bernal-Quiros M, Wu YY, Alarcon-Riquelme ME, Castillejo-Lopez C. BANK1 and BLK act through phospholipase C gamma 2 in B-cell signaling. PLoS One. 2013;8(3):e59842. Epub 2013/04/05. doi: 10.1371/journal.pone.0059842. PubMed PMID: 23555801; PubMed Central PMCID: PMCPMC3608554.

2. Draber P, Vonkova I, Stepanek O, Hrdinka M, Kucova M, Skopcova T, et al. SCIMP, a transmembrane adaptor protein involved in major histocompatibility complex class II signaling. Mol Cell Biol. 2011;31(22):4550-62. Epub 2011/09/21. doi: 10.1128/MCB.05817-11. PubMed PMID: 21930792; PubMed Central PMCID: PMCPMC3209250.

3. Kralova J, Fabisik M, Pokorna J, Skopcova T, Malissen B, Brdicka T. The Transmembrane Adaptor Protein SCIMP Facilitates Sustained Dectin-1 Signaling in Dendritic Cells. J Biol Chem. 2016;291(32):16530-40. Epub 2016/06/12. doi: 10.1074/jbc.M116.717157. PubMed PMID: 27288407; PubMed Central PMCID: PMCPMC4974369.

4. Weekes MP, Antrobus R, Talbot S, Hor S, Simecek N, Smith DL, et al. Proteomic plasma membrane profiling reveals an essential role for gp96 in the cell surface expression of LDLR family members, including the LDL receptor and LRP6. J Proteome Res. 2012;11(3):1475-84. Epub 2012/02/02. doi: 10.1021/pr201135e. PubMed PMID: 22292497; PubMed Central PMCID: PMCPMC3292266.

5. Dominguez-Soto A, Aragoneses-Fenoll L, Martin-Gayo E, Martinez-Prats L, Colmenares M, Naranjo-Gomez M, et al. The DC-SIGN-related lectin LSECtin mediates antigen capture and pathogen binding by human myeloid cells. Blood. 2007;109(12):5337-45. Epub 2007/03/07. doi: 10.1182/blood-2006-09-048058. PubMed PMID: 17339424.

6. Weiss MJ, Henthorn PS, Lafferty MA, Slaughter C, Raducha M, Harris H. Isolation and characterization of a cDNA encoding a human liver/bone/kidney-type alkaline phosphatase. Proc Natl Acad Sci U S A. 1986;83(19):7182-6. Epub 1986/10/01. doi: 10.1073/pnas.83.19.7182. PubMed PMID: 3532105; PubMed Central PMCID: PMCPMC386679.

7. Bergin DA, Reeves EP, Meleady P, Henry M, McElvaney OJ, Carroll TP, et al. alpha-1 Antitrypsin regulates human neutrophil chemotaxis induced by soluble immune complexes and IL-8. J Clin Invest. 2010;120(12):4236-50. Epub 2010/11/10. doi: 10.1172/JCI41196. PubMed PMID: 21060150; PubMed Central PMCID: PMCPMC2993580.

8. Kamohara H, Matsuyama W, Shimozato O, Abe K, Galligan C, Hashimoto S, et al. Regulation of tumour necrosis factor-related apoptosis-inducing ligand (TRAIL) and TRAIL receptor expression in human neutrophils. Immunology. 2004;111(2):186-94. Epub 2004/03/19. doi: 10.1111/j.0019-2805.2003.01794.x. PubMed PMID: 15027904; PubMed Central PMCID: PMCPMC1782413.

9. Elghetany MT. Surface antigen changes during normal neutrophilic development: a critical review. Blood Cells Mol Dis. 2002;28(2):260-74. Epub 2002/06/18. PubMed PMID: 12064921.

10. Starr AE, Bellac CL, Dufour A, Goebeler V, Overall CM. Biochemical characterization and N-terminomics analysis of leukolysin, the membrane-type 6 matrix metalloprotease (MMP25): chemokine and vimentin cleavages enhance cell migration and macrophage phagocytic activities. J Biol Chem. 2012;287(16):13382-95. Epub 2012/03/01. doi: 10.1074/jbc.M111.314179. PubMed PMID: 22367194; PubMed Central PMCID: PMCPMC3339980.

11. Montell C. Mg2+ homeostasis: the Mg2+nificent TRPM chanzymes. Curr Biol. 2003;13(20):R799-801. Epub 2003/10/17. PubMed PMID: 14561419.

12. Nadolni W, Zierler S. The Channel-Kinase TRPM7 as Novel Regulator of Immune System Homeostasis. Cells. 2018;7(8). Epub 2018/08/22. doi: 10.3390/cells7080109. PubMed PMID: 30126133; PubMed Central PMCID: PMCPMC6115979.

13. Coakley RJ, Taggart C, Greene C, McElvaney NG, O'Neill SJ. Ambient pCO2 modulates intracellular pH, intracellular oxidant generation, and interleukin-8 secretion in human neutrophils. J Leukoc Biol. 2002;71(4):603-10. Epub 2002/04/03. PubMed PMID: 11927646.

14. Barker H, Aaltonen M, Pan P, Vahatupa M, Kaipiainen P, May U, et al. Role of carbonic anhydrases in skin wound healing. Exp Mol Med. 2017;49(5):e334. Epub 2017/05/20. doi: 10.1038/emm.2017.60. PubMed PMID: 28524177; PubMed Central PMCID: PMCPMC5454449.

15. Zhou W, Zhao Q, Sutton R, Cumming H, Wang X, Cerruti L, et al. The role of p22 NF-E4 in human globin gene switching. J Biol Chem. 2004;279(25):26227-32. Epub 2004/04/16. doi: 10.1074/jbc.M402191200. PubMed PMID: 15084587.

16. Gilbert C, Levasseur S, Desaulniers P, Dusseault AA, Thibault N, Bourgoin SG, et al. Chemotactic factor-induced recruitment and activation of Tec family kinases in human neutrophils. II. Effects of LFM-A13, a specific Btk inhibitor. J Immunol. 2003;170(10):5235-43. Epub 2003/05/08. PubMed PMID: 12734372.

17. Xu K, Cooney KA, Shin EY, Wang L, Deppen JN, Ginn SC, et al. Adenosine from a biologic source regulates neutrophil extracellular traps (NETs). J Leukoc Biol. 2019. Epub 2019/03/26. doi: 10.1002/JLB.3VMA0918-374R. PubMed PMID: 30907983.

18. Liu YW, Yang T, Zhao L, Ni Z, Yang N, He F, et al. Activation of Adenosine 2A receptor inhibits neutrophil apoptosis in an autophagy-dependent manner in mice with systemic inflammatory response syndrome. Sci Rep. 2016;6:33614. Epub 2016/09/21. doi: 10.1038/srep33614. PubMed PMID: 27647162; PubMed Central PMCID: PMCPMC5028892.

19. Canela L, Lujan R, Lluis C, Burgueno J, Mallol J, Canela EI, et al. The neuronal Ca(2+) -binding protein 2 (NECAB2) interacts with the adenosine A(2A) receptor and modulates the cell surface expression and function of the receptor. Mol Cell Neurosci. 2007;36(1):1-12. Epub 2007/08/11. doi: 10.1016/j.mcn.2007.05.007. PubMed PMID: 17689978.

20. Jiang S, Zhang M, Sun J, Yang X. Casein kinase 1alpha: biological mechanisms and theranostic potential. Cell Commun Signal. 2018;16(1):23. Epub 2018/05/26. doi: 10.1186/s12964-018-0236-z. PubMed PMID: 29793495; PubMed Central PMCID: PMCPMC5968562.

21. Maecker HT, McCoy JP, Nussenblatt R. Standardizing immunophenotyping for the Human Immunology Project. Nat Rev Immunol. 2012;12(3):191-200. Epub 2012/02/22. doi: 10.1038/nri3158. PubMed PMID: 22343568; PubMed Central PMCID: PMCPMC3409649.

22. Pitard V, Roumanes D, Lafarge X, Couzi L, Garrigue I, Lafon ME, et al. Long-term expansion of effector/memory Vdelta2-gammadelta T cells is a specific blood signature of CMV infection. Blood. 2008;112(4):1317-24. Epub 2008/06/10. doi: 10.1182/blood-2008-01-136713. PubMed PMID: 18539896; PubMed Central PMCID: PMCPMC2515135.

23. Farber DL, Yudanin NA, Restifo NP. Human memory T cells: generation, compartmentalization and homeostasis. Nat Rev Immunol. 2014;14(1):24-35. Epub 2013/12/18. doi: 10.1038/nri3567. PubMed PMID: 24336101; PubMed Central PMCID: PMCPMC4032067.

24. Angelo LS, Banerjee PP, Monaco-Shawver L, Rosen JB, Makedonas G, Forbes LR, et al. Practical NK cell phenotyping and variability in healthy adults. Immunol Res. 2015;62(3):341-56. Epub 2015/05/28. doi: 10.1007/s12026-015-8664-y. PubMed PMID: 26013798; PubMed Central PMCID: PMCPMC4470870.

25. Boyette LB, Macedo C, Hadi K, Elinoff BD, Walters JT, Ramaswami B, et al. Phenotype, function, and differentiation potential of human monocyte subsets. PLoS One. 2017;12(4):e0176460. Epub 2017/04/27. doi: 10.1371/journal.pone.0176460. PubMed PMID: 28445506; PubMed Central PMCID: PMCPMC5406034.

26. Verreck FA, de Boer T, Langenberg DM, van der Zanden L, Ottenhoff TH. Phenotypic and functional profiling of human proinflammatory type-1 and anti-inflammatory type-2 macrophages in response to microbial antigens and IFN-gamma- and CD40L-mediated costimulation. J Leukoc Biol. 2006;79(2):285-93. Epub 2005/12/07. doi: 10.1189/jlb.0105015. PubMed PMID: 16330536.

27. Rey-Giraud F, Hafner M, Ries CH. In vitro generation of monocyte-derived macrophages under serum-free conditions improves their tumor promoting functions. PLoS One. 2012;7(8):e42656. Epub 2012/08/11. doi: 10.1371/journal.pone.0042656. PubMed PMID: 22880072; PubMed Central PMCID: PMCPMC3412794.

28. Pang DJ, Neves JF, Sumaria N, Pennington DJ. Understanding the complexity of gammadelta T-cell subsets in mouse and human. Immunology. 2012;136(3):283-90. Epub 2012/03/06. doi: 10.1111/j.1365-2567.2012.03582.x. PubMed PMID: 22385416; PubMed Central PMCID: PMCPMC3385028.

29. Farag SS, Caligiuri MA. Human natural killer cell development and biology. Blood Rev. 2006;20(3):123-37. Epub 2005/12/21. doi: 10.1016/j.blre.2005.10.001. PubMed PMID: 16364519.

30. Law CW, Chen Y, Shi W, Smyth GK. voom: Precision weights unlock linear model analysis tools for RNA-seq read counts. Genome Biol. 2014;15(2):R29. Epub 2014/02/04. doi: 10.1186/gb-2014-15-2-r29. PubMed PMID: 24485249; PubMed Central PMCID: PMCPMC4053721.

31. Zhang B, Horvath S. A general framework for weighted gene co-expression network analysis. Stat Appl Genet Mol Biol. 2005;4:Article17. Epub 2006/05/02. doi: 10.2202/1544-6115.1128. PubMed PMID: 16646834.

32. Langfelder P, Horvath S. WGCNA: an R package for weighted correlation network analysis. BMC Bioinformatics. 2008;9:559. Epub 2008/12/31. doi: 10.1186/1471-2105-9-559. PubMed PMID: 19114008; PubMed Central PMCID: PMCPMC2631488.

33. Lambert SA, Jolma A, Campitelli LF, Das PK, Yin Y, Albu M, et al. The Human Transcription Factors. Cell. 2018;172(4):650-65. Epub 2018/02/10. doi: 10.1016/j.cell.2018.01.029. PubMed PMID: 29425488.
